# Dual-Target Compounds against Type 2 *Diabetes Mellitus*: Proof of Concept for Sodium Dependent Glucose Transporter (SGLT) and Glycogen Phosphorylase (GP) Inhibitors

**DOI:** 10.3390/ph14040364

**Published:** 2021-04-15

**Authors:** Ádám Sipos, Eszter Szennyes, Nikolett Éva Hajnal, Sándor Kun, Katalin E. Szabó, Karen Uray, László Somsák, Tibor Docsa, Éva Bokor

**Affiliations:** 1 Department of Medical Chemistry, Faculty of Medicine, University of Debrecen, Egyetem tér 1, H-4032 Debrecen, Hungary; sipos.adam@med.unideb.hu (Á.S.); karen.uray@med.unideb.hu (K.U.); 2 Doctoral School of Molecular Medicine, University of Debrecen, Egyetem tér 1, H-4032 Debrecen, Hungary; 3 Department of Organic Chemistry, University of Debrecen, POB 400, H-4002 Debrecen, Hungary; szeszterke11@gmail.com (E.S.); hajnalnikoletteva@gmail.com (N.É.H.); kun.sandor@science.unideb.hu (S.K.); szabo.erzsebet.katalin@science.unideb.hu (K.E.S.)

**Keywords:** *C*-glucosyl heterocycle, imidazole, thiazole, 1,2,3- and 1,2,4-triazole, 1,2,4- and 1,3,4-oxadiazole, pyrimidine, sodium dependent glucose cotransporter, glycogen phosphorylase, dual-target inhibitor

## Abstract

A current trend in the quest for new therapies for complex, multifactorial diseases, such as diabetes mellitus (DM), is to find dual or even multi-target inhibitors. In DM, the sodium dependent glucose cotransporter 2 (SGLT2) in the kidneys and the glycogen phosphorylase (GP) in the liver are validated targets. Several (β-D-glucopyranosylaryl)methyl (het)arene type compounds, called gliflozins, are marketed drugs that target SGLT2. For GP, low nanomolar glucose analogue inhibitors exist. The purpose of this study was to identify dual acting compounds which inhibit both SGLTs and GP. To this end, we have extended the structure-activity relationships of SGLT2 and GP inhibitors to scarcely known (*C*-β-D-glucopyranosylhetaryl)methyl arene type compounds and studied several (*C*-β-D-glucopyranosylhetaryl)arene type GP inhibitors against SGLT. New compounds, such as 5-arylmethyl-3-(β-D-glucopyranosyl)-1,2,4-oxadiazoles, 5-arylmethyl-2-(β-D-glucopyranosyl)-1,3,4-oxadiazoles, 4-arylmethyl-2-(β-D-glucopyranosyl)pyrimidines and 4(5)-benzyl-2-(β-D-glucopyranosyl)imidazole were prepared by adapting our previous synthetic methods. None of the studied compounds exhibited cytotoxicity and all of them were assayed for their SGLT1 and 2 inhibitory potentials in a SGLT-overexpressing TSA201 cell system. GP inhibition was also determined by known methods. Several newly synthesized (*C*-β-D-glucopyranosylhetaryl)methyl arene derivatives had low micromolar SGLT2 inhibitory activity; however, none of these compounds inhibited GP. On the other hand, several (*C*-β-D-glucopyranosylhetaryl)arene type GP inhibitor compounds with low micromolar efficacy against SGLT2 were identified. The best dual inhibitor, 2-(β-D-glucopyranosyl)-4(5)-(2-naphthyl)-imidazole, had a K_i_ of 31 nM for GP and IC_50_ of 3.5 μM for SGLT2. This first example of an SGLT-GP dual inhibitor can prospectively be developed into even more efficient dual-target compounds with potential applications in future antidiabetic therapy.

## 1. Introduction

Diabetes mellitus (DM) is a highly prevalent disease, affecting an estimated 9.3% of the adult population worldwide [1]. More than 463 million adults have diabetes and 352 million of them are middle-aged working people. In 2019, more than 50% of the population had undiagnosed diabetes [1]. Diabetics have an increased risk of complications, including retinopathy, blindness, kidney failure, cardiovascular disease, neuropathy, stroke, and loss of toes, feet, or legs. 

Type 2 diabetes (non-insulin-dependent or T2DM) is a chronic disease characterized by hyperglycaemia resulting from defects in insulin secretion and/or action and increased hepatic glucose output [2,3]. More than 90% of diabetic patients have T2DM. Thus, T2DM represents a major public health problem, mainly due to complications. In the absence of a causal cure, the management of T2DM is focused on lowering blood glucose levels to prevent and delay the onset of diabetic complications. However, glycaemic control is often difficult to achieve with oral antidiabetic agents [4]. 

Current anti-hyperglycaemic agents target the pathophysiological defects of T2DM, including appetite control and nutrient absorption and excretion. Several well-defined types of hypoglycaemic agents are available [5] with unique pharmacological properties. If adequate control cannot be obtained with the use of a single agent, combination therapy is recommended using multiple drugs with different mechanisms of action [6]. Despite the armoury of hypoglycaemic drugs, glycaemic control is still problematic in diabetic patients and the development of novel drugs is necessary to diminish morbidity, lessen complications, and improve quality of life. 

Beyond combination therapies, a new approach in drug design against complex diseases is the quest for dual- or multi-target compounds that act on more than one biological macromolecule [7]. In this respect, the fields of cancer [8] and neurodegeneration [9] have been surveyed. In the context of T2DM, the development of such multi-target drugs has recently been reviewed [10,11,12] to highlight agonists acting on incretin, glucagon systems, and peroxisome proliferation activated receptors, just to mention a few. Another interesting example is the dual inhibition of aldose reductase (AR, IC_50_ 0.056 μM) and protein tyrosine phosphatase 1B (PTP1B, IC_50_ 32.5 μM) by a (5-arylidene-4-oxo-2-thioxothiazolidin-3-yl)acetic acid derivative with the potential to treat T2DM [13]. This class of dual inhibitors is under further development [14,15,16].

The newest antidiabetic drugs on the market are sodium dependent glucose cotransporter 2 (SGLT2) inhibitors, termed gliflozins [17,18,19]. Gliflozins decrease renal reabsorption of glucose and increase glucose concentration in the vacated urine, thereby leading to decreased blood sugar levels in diabetic patients [20,21]. Gliflozins were developed based on the long-known hypoglycaemic effects of the natural product phlorizin (**1**, Table 1) [22]. Currently, there are seven approved SGLT2 inhibitors available as oral antidiabetics for use in T2DM [23]. Gliflozins are used alone or in combination with other oral antidiabetic drugs [24,25,26,27]. 

The most used SGLT2 inhibitors are dapagliflozin (**2**), empagliflozin (**3**), canagliflozin (**4**), and ipragliflozin (**5**) [28]. These antidiabetic drugs significantly reduce deaths and hospitalization among individuals with T2DM and established cardiovascular disease. A meta-analysis of many cohorts treated with SGLT2 inhibitors [29,30,31] demonstrated that T2DM patients treated with gliflozins had a lower rate of comorbidities (coronary syndrome, heart disease, heart failure, etc.), including a primary composite cardiovascular outcome, and decreased death from any cause when the study drug was added to standard care [32]. However, diabetic ketoacidosis is a serious complication of diabetes caused by low insulin levels. Rare cases of this condition, including life-threatening ones, have occurred in patients taking gliflozins for T2DM. A number of these cases have been atypical, with patients who didn’t have blood sugar levels as high as expected [33]. The European Medicines Agency (EMA) and the US Food and Drug Administration (FDA) have issued recommendations to minimize diabetic ketoacidosis risk in patients taking SGLT2 inhibitors. Sotagliflozin (**6**), a dual SGLT2 and SGLT1 inhibitor, has been approved by EMA, but not FDA, as an adjunct to insulin in T1DM [34].

**Figure 1:**
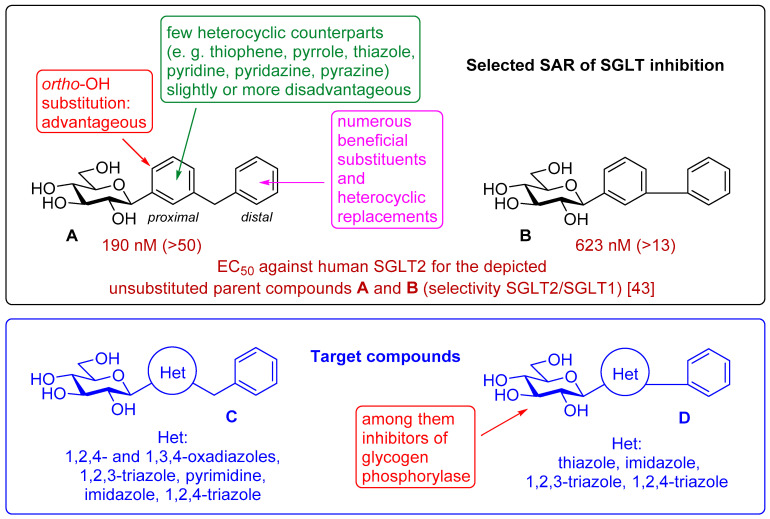
Excerpt of structure-activity relationships of SGLT inhibitors and the target compounds in this study.

Chemically, the marketed gliflozins are (β-D-glucopyranosylaryl)methyl (het)arenes. This scaffold is the result of many rounds of iterations, including several *O*- and *N*-glycosidic compounds, to improve pharmacodynamic and pharmacokinetic properties of phlorizin to finally arrive at the *C*-glucosylic structure [19,20,39,40,41,42]. After the fortuitous discovery of the highly beneficial meta arrangement of the glycosyl and the benzyl moieties on the proximal aromatic ring (Figure 1, **A**) [43], this skeleton has become the lead structure for further structure-activity relationship (SAR) studies to modify the sugar part and replace or substitute both aromatic rings and the methylene linker between them [19,20,44,45,46].

While the exchange of the distal aromatic moiety was rather extensively investigated [19,20,44,45,46], resulting in the marketed canagliflozin **4** and ipragliflozin **5**, fewer studies targeted the replacement of the proximal benzene unit by hetarenes (Figure 1) [19,21]. The known structures include thiophene [38,47,48], pyrrole [38], thiazole [49], pyridine [38], pyridazine [49], and pyrazine [38] rings, which show IC_50_ values from the low nM to the low μM range. The IC_50_-s are strongly dependent on the heterocyclic moiety. The introduction of a phenolic OH into the *ortho* position of the proximal ring proved advantageous in several cases, indicating a possible role for a hydrogen bond-forming group in that region [38,50,51,52,53,54]. 

As the constitution of the heterocycle may have an essential impact on the efficiency of the molecules against SGLTs, further replacements of the proximal benzene ring were envisaged (see target compounds **C** in Figure 1). The foreseen imidazole and 1,2,4-triazole derivatives may form H-bonds close to the position of the above phenolic OH. Furthermore, taking into account that the glucosyl biphenyl derivative **B** also inhibited the SGLTs [43], the study of compounds **D** was also envisaged. Since many of the **D** type derivatives proved to be potent inhibitors of glycogen phosphorylases (GP) [21,55,56], another validated target in combatting T2DM, these compounds may prove the dual-target concept for SGLT-GP which, to the best of our knowledge, has never yet been investigated. 

Thus, the purpose of this study is to identify new SGLT2 inhibitors with dual activity at GP by characterizing their inhibitory properties towards both proteins. Beginning with the basic structure of clinically available SGLT2 inhibitors, the proximal aromatic group was exchanged with various heterocyclic groups (Figure 1, **C**). Cell lines stably transfected with SGLT1 and 2 were used to compare the inhibitory properties of these newly synthesized compounds with dapagliflozin and phlorizin, and to reveal selectivity between the two SGLTs. First, the activities of the type **C** compounds were examined against the SGLT and GP proteins, then the inhibitory effects of previously published GP inhibitors (Figure 1, **D**) were tested against SGLTs using the new functional assay system. Dual active compounds may serve as lead structures for further dual inhibitor design.

## 2. Results and Discussion

### 2.1. Syntheses

**Scheme 1:**
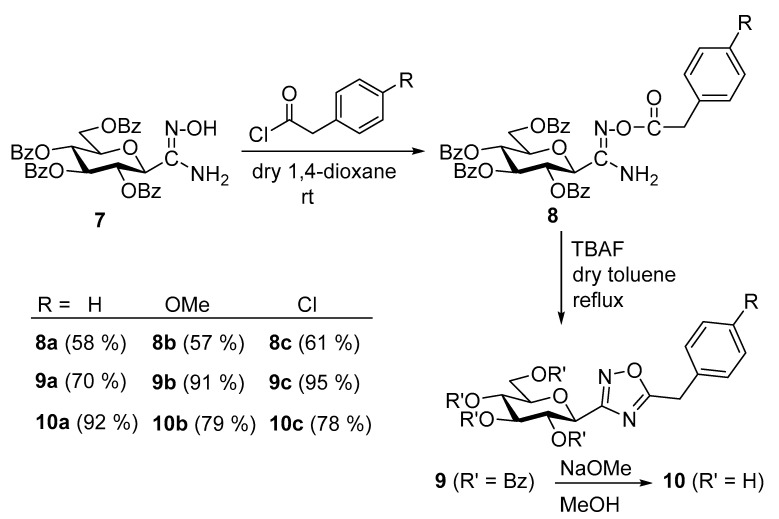
Synthesis of 5-arylmethyl-3-(β-D-glucopyranosyl)-1,2,4-oxadiazoles.

The synthesis of 5-arylmethyl-3-(β-D-glucopyranosyl)-1,2,4-oxadiazoles was accomplished by adaptation of a published procedure [57,58]. Thus, *O*-perbenzoylated *C*-(β-D-glucopyranosyl)formamidoxime **7** [57,58] was *O*-acylated with the corresponding phenylacetyl chlorides and the resulting *O*-acyl-amidoximes **8** were cyclized in the presence of TBAF to get compounds **9** in good yields. *O*-Debenzoylation of **9** was carried out by the Zemplén method, resulting in the test compounds **10** in excellent yields (Scheme 1).

**Scheme 2:**
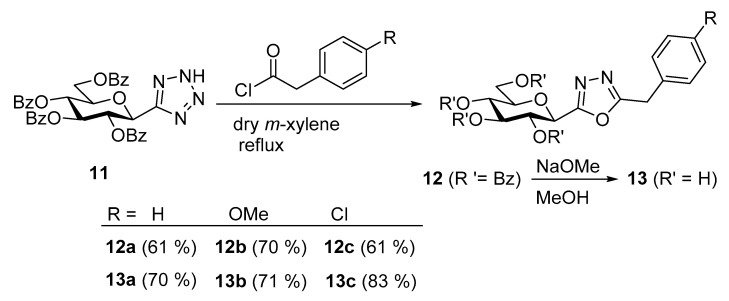
Synthesis of 5-arylmethyl-2-(β-D-glucopyranosyl)-1,3,4-oxadiazoles.

Based on literature analogies [59,60,61], the isomeric 5-arylmethyl-2-(β-D-glucopyranosyl)-1,3,4-oxadiazoles **13** were synthesized starting from the easily available 5-(2,3,4,6-tetra-*O*-benzoyl-β-D-glucopyranosyl)tetrazole **11** [59,61]. The ring-transformation reactions upon treatment of tetrazole **11** with phenylacetyl chlorides afforded the protected oxadiazoles **12**, which were *O*-debenzoylated by transesterification to give the final products **13** in good yields (Scheme 2).

**Scheme 3:**
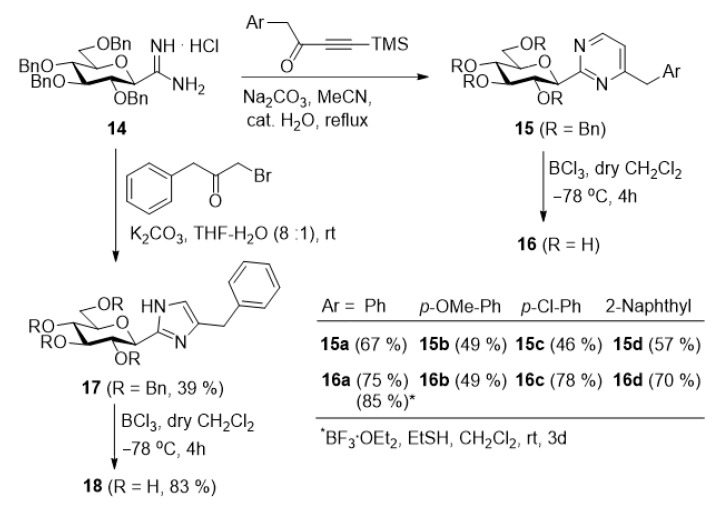
Synthesis of 4-arylmethyl-2-(β-D-glucopyranosyl)pyrimidines and 4(5)-benzyl-2-(β-D-glucopyranosyl)imidazole.

In recent papers [62,63], the *O*-perbenzylated *C*-(β-D-glucopyranosyl)formamidine hydrochloride **14** [64,65] was shown to be a suitable precursor for Pinner-type synthesis of variously substituted 2-(β-D-glucopyranosyl)pyrimidines. Based on these preliminaries, the preparation of 4-arylmethyl-2-(β-D-glucopyranosyl)pyrimidines was also envisaged by base-mediated cyclocondensations of **14**. To this end, amidine salt **14** was treated with the appropriate trimetylsilylated alkynyl ketones in the presence of Na_2_CO_3_, resulting in the desired pyrimidines **15** in acceptable yields (Scheme 3). *O*-Debenzylation was then performed by the reaction of **15** with BCl_3_, affording the test compounds **16** in good yields. Removal of the benzyl protecting groups of **15a** was also carried out using ethanethiol in the presence of BF_3_·OEt_2_. Of note, a somewhat better yield of **16a** was observed under these conditions; however, the complete deprotection required a significantly longer reaction time compared to the former method (3 days vs. 4 h).

Following our earlier elaborated method [64], the preparation of 4(5)-benzyl-2-(β-D-glucopyranosyl)imidazole **18** was also accomplished from **14** by its ring-closure with 1-bromo-3-phenylpropan-2-one under basic conditions to give **17** followed by *O*-deprotection of the sugar moiety.

### 2.2. Biological Studies

**Figure 2:**
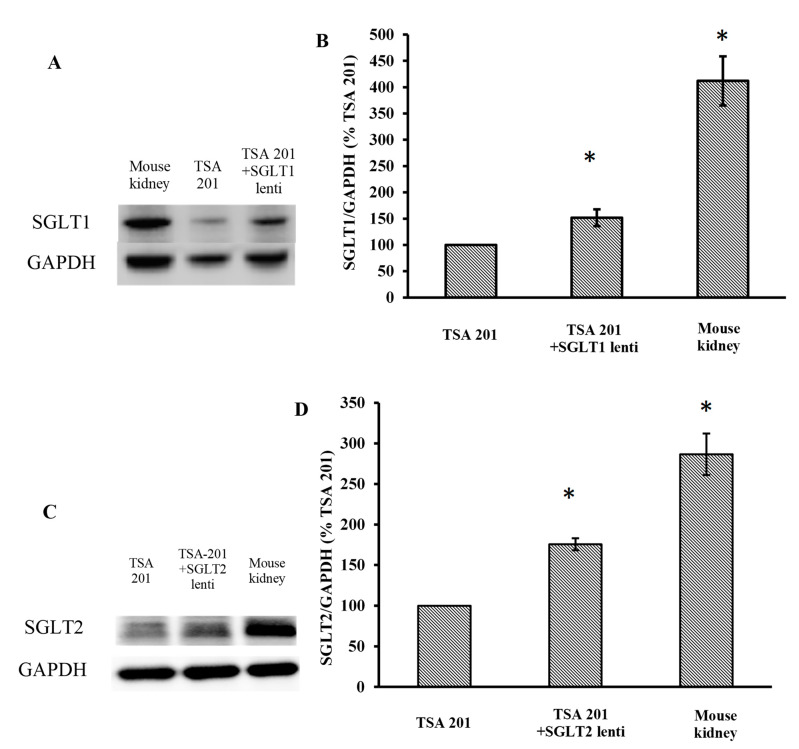
Expression of SGLT1 (**A**,**B**) and SGLT2 (**C**,**D**) in transfected TSA201 cells and mouse kidney by Western blot. GAPDH was used to normalize for protein loading. The results were quantified by BIORAD Image Lab 5.2.1 software. (* *p* < 0.05 vs. TSA 201, *n* = 3 independent experiment per group). The kidney is a positive control to validate our SGLT1 and 2 antibodies. Full blot pictures are shown in Figures S1 and S2 in the Supplementary Information.

**Figure 3:**
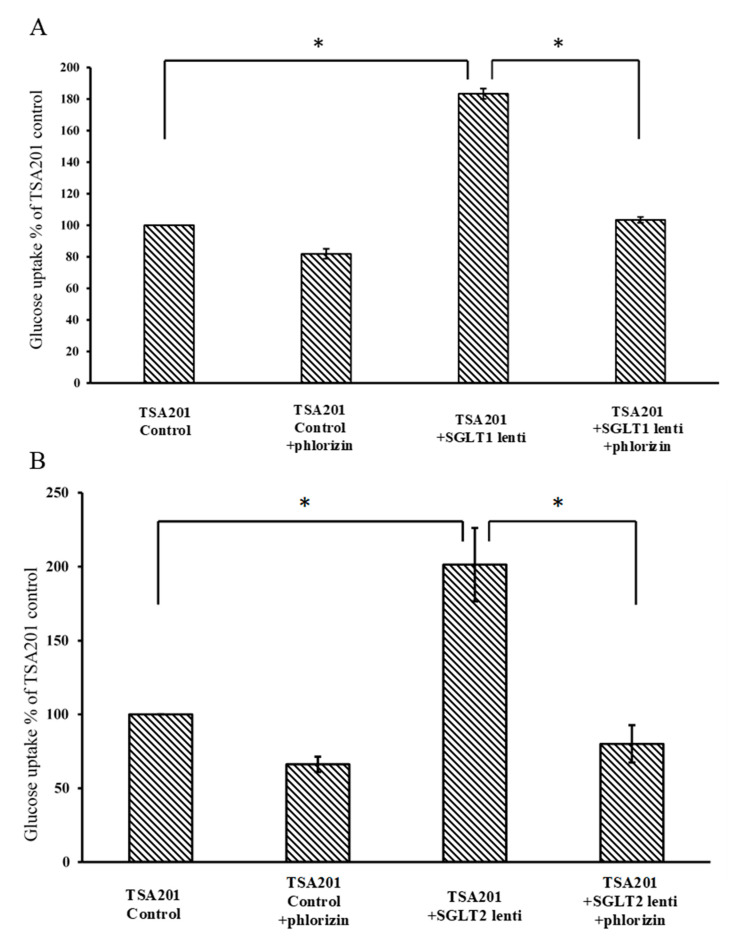
Glucose uptake assay of control and SGLT1 (**A**) and SGLT2 (**B**)-transfected TSA201 cells. Cells were treated with 2-NBDG (2-deoxy-2-[(7-nitro-2,1,3-benzoxadiazol-4-yl)amino]-D-glucose) in the presence or absence of phlorizin. Glucose uptake was increased significantly in SGLT1 and SGLT2-transfected cells compared with control TSA201 cells. Phlorizin significantly decreased glucose uptake in both control and SGLT1 and SGLT2-transfected cells. (*n* = 3 per group; * *p* < 0.05).

**Figure 4:**
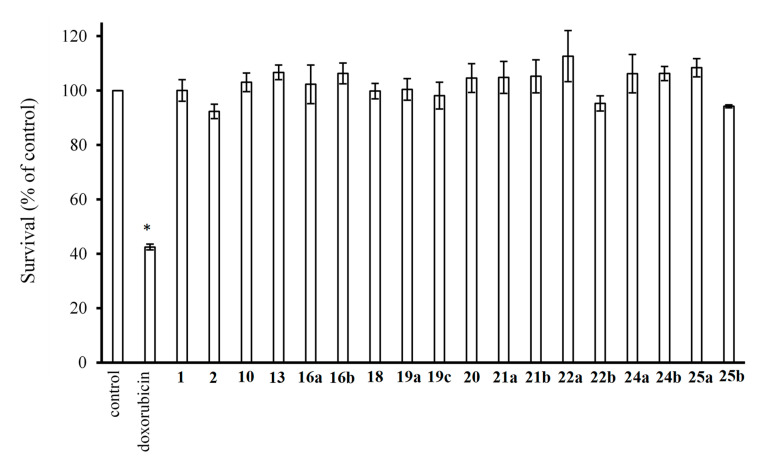
Cytotoxicity of the studied compounds (1000 μM). Cell survival was measured by CyQUANT assay. Data were normalized to control cells treated with vehicle only. Doxorubicin (2 ng/mL) was used as a negative control (* *p* < 0.05 vs. control; *n* = 3 per group).

To identify the inhibitory effects of the test compounds, new cell lines were created that stably overexpress SGLT1 and 2 using a lentiviral system. As shown in Figure 2A,C, SGLT1 and 2 are upregulated after lentiviral transfection (full blot pictures are shown in Figures S1 and S2 in the Supplementary Information). Figure 3 shows that glucose uptake is significantly increased in the TSA201 cells overexpressing SGLT1 and 2 compared to non-transfected cells. To determine the suitability of the new cell lines for assaying SGLT1 and 2 inhibitors, the known SGLT1 and 2 inhibitor, phlorizin **1,** and the patented SGLT2 inhibitor, dapagliflozin **2**, were tested. The data for phlorizin is shown in Figure 3. Phlorizin attenuated the increase in glucose uptake induced by overexpression of SGLT1 and 2, demonstrating the specificity of the assay. None of the compounds exhibited cytotoxicity, as shown in Figure 4. These data were utilized to generate dose-response curves for the calculation of the IC_50_s shown in Table 2 and Table 3. Examples of dose-response curves for dapagliflozin and compounds **22b** and **24b** are shown in the Supplementary Figure S3.

Tests carried out with the newly synthesized and some previously prepared arylmethyl substituted glucosyl heterocycles (**10**, **13**, **16**, **18**–**20)** revealed some new SGLT2 inhibitors with IC_50_-s in the micromolar range (Table 2). Although the best inhibitors of this set (**10a**, **13a**, **18**, and **19c**) showed remarkably weaker inhibitory potencies than the known *C*-glucosyl arene type inhibitor **2**, their activities exceeded that of phlorizin (**1**). Nevertheless, this study confirmed earlier observations for the *C*-glucosyl (het)arene type SGLT2 inhibitors demonstrating that replacement of the proximal aromatic ring with heterocycles leads to decreased inhibitory efficacy, which is strongly dependent on the structure of the heteroring [19,21].

The SGLT2 inhibitory effect of the benzyl substituted oxadiazoles indicated that the constitution of the oxadiazole ring did not significantly affect the inhibitory potential (Table 2, **10a** vs. **13a**). However, replacement of the heterocyclic oxygen by a H-bond donor NH group led to a 20-30-fold weakening in the inhibition by the corresponding 1,2,4-triazole (**19a** vs. **10a** and **13a**). The introduction of a phenyl ring to the N1-atom of the 1,2,4-triazole resulted in significant improvement in the efficacy (**19c** vs. **19a**), while attaching a hydroxyethyl group to the same position caused complete loss of the inhibition (**19a** vs. **19b**). Compared to 1,2,4-triazole **19a** imidazole **18**, which also contained an NH group, proved to be ~20 times more effective. The role of the NH group seems contradictory, since compound **18** is stronger but **19a** is a weaker inhibitor. We speculate that this might be the result of three possible tautomeric forms of the 1,2,4-triazole **19a**, from which only one may bind to the protein, while for the imidazole **18** the formally existing tautomers are essentially the same due to a probable protonation in the biological medium [66].

Similar to other classes of SGLT2 inhibitors, the inhibition highly depends on substitution of the distal aromatic ring of the aglycon part. In the case of the 1,2,4- and 1,3,4-oxadiazoles, only the unsubstituted derivatives (Table 2, **10a** and **13a**) showed inhibitory effects, while the introduction of a methoxy or a chloro group into the *para* position of the benzene ring, such as in **10b** and **13b** and **10c** and **13c**, respectively, led to complete loss of the inhibition. In the pyrimidine series, not only the benzyl (**16a**), but also the 4-methoxybenzyl (**16b**) derivative, displayed inhibitory activity in the micromolar range. In contrast, compound **16c**, which has a 4-chlorobenzyl residue, remained inactive. Replacement of the distal benzene ring by a 2-naphthyl moiety resulted in a non-inhibitory molecule **16d**. None of the compounds listed in Table 2 had remarkable inhibitory effects against rabbit muscle GPb enzyme in the concentration tested.

Next, a series of known GP inhibitors **21*–*25** (Table 3) synthesized and investigated in our group [68,69,70,71,72,73] were assayed against SGLTs. The compounds were selected from non-inhibitory ones to low nanomolar GP inhibitors. Former GP inhibition studies revealed the essential role of the heterocyclic linker between the glucose unit and the distal aromatic moiety [21,55,56]. With respect to the latter aromatic group, the 2-naphthyl substituted derivatives **21b**–**25b** surpassed the phenyl substituted ones **21a**–**25a** by a factor of approx. 10 in most cases. The SGLT assay gratifyingly indicated low micromolar inhibitions for many of these compounds. Though thiazoles **23** proved inefficient towards both transporters in the studied concentration, the rest of the compounds had varying effects strongly indicating the substantial role of the heterocyclic linker in this biomolecular interaction. 

Most of the compounds in Table 3 inhibited SGLT2 more than SGLT1 with a selectivity range of ~2–27. There were two exceptions to this trend: compounds **22b** and **25b** (both with a 2-naphthyl substituent) showed some preference for SGLT1 inhibition. In two comparisons, **21a–21b** and **24a–24b** the 2-naphthyl derivatives were stronger inhibitors of SGLT2 than SGLT1 by a factor of ~7 and ~26, respectively. The 2-(β-D-glucopyranosyl)-4(5)-(2-naphthyl)-imidazole **24b**, with a 3.5 μM IC_50_ towards SGLT2, emerged as the best inhibitor. Taking into account its nanomolar inhibition of GP, **24b** represents a dual-targeting compound, which can be a starting point for further antidiabetic studies in this direction. The SGLT inhibitors are highly sensitive to the substitution pattern of the aglycon as outlined in the introduction. On the other hand, the substituent effects in glucosyl heterocycles on GP inhibition are relatively unknown [56]. Thus, extensive computational and synthetic work will be necessary to optimize the dual-target efficiency. Our present efforts are extending in these directions.

## 3. Materials and Methods

### 3.1. Syntheses

#### 3.1.1. General Methods

Melting points were measured on a Kofler hot-stage and the values are uncorrected. Optical rotations were determined on a P-2000 polarimeter (Jasco, Easton, MD, USA) at rt. NMR spectra were recorded with DRX360 (360/90 MHz for ^1^H/^13^C) and DRX400 (400/100 MHz for ^1^H/^13^C) spectrometers (Bruker, Karlsruhe, Germany). Chemical shifts are referenced to internal Me_4_Si (^1^H) or the residual solvent signals (^13^C). To avoid ambiguities in assignment of the NMR signals sugar protons and carbons are marked by primed numbers. HRMS spectra were obtained by a Bruker maXis II spectrometer using the electrospray ionization technique. TLC was performed on DC Alurolle Kieselgel 60 F_254_ (Merck, Darmstadt, Germany) and the plates were visualized by gentle heating. Column chromatography was carried out by applying Kieselgel 60 (particle size 63–200 µm; Molar Chemicals, Halásztelek, Hungary) silica gel. Anhydrous solvents were obtained by standard distillation methods. CH_2_Cl_2_, CHCl_3_, CH_3_CN (4 Å molecular sieves), toluene, and *m*-xylene (sodium wires) were distilled from P_4_O_10_ and stored over the indicated drying agents. MeOH was distilled over Mg turnings and iodine. Distillation of 1,4-dioxane was performed from sodium benzophenone ketyl and the solvent was stored over sodium wires. *C*-(2,3,4,6-Tetra-*O-*benzoyl-β-D-glucopyranosyl)formamidoxime (**7**) [58], 5-(2,3,4,6-tetra-*O-*benzoyl-β-D-glucopyranosyl)tetrazole (**11**) [61], *C*-(2,3,4,6-tetra-*O*-benzyl-β-D-glucopyranosyl)formamidine hydrochloride (**14**) [64,65], and the 1-substituted-4-(trimethylsilyl)but-3-in-4-ones (substituent: phenyl [74], 4-chlorophenyl [75], 4-methoxyphenyl [76], and 2-naphthyl [77]) were prepared according to the respective published procedures. 

#### 3.1.2. General Procedure 1 for the Synthesis of *O*-acyl-*C*-(2,3,4,6-Tetra-*O*-Benzoyl-β-D-Glucopyranosyl)Formamidoximes **8**

To a solution of *C*-(2,3,4,6-tetra-*O*-benzoyl-β-D-glucopyranosyl)formamidoxime (**7**) in anhydrous 1,4-dioxane (3 mL/100 mg substrate), the corresponding acid chloride (1.1 equiv.) was added and the reaction mixture was stirred at rt. After the disappearance of the starting amidoxime **7**, judged by TLC (EtOAc-hexane 1:2), the solvent was removed by diminished pressure and the crude product was purified by column chromatography. 

#### 3.1.3. General Procedure 2 for the Synthesis of 5-(4-Substituted-Benzyl)-3-(2,3,4,6-Tetra-*O*-Benzoyl-β-D-Glucopyranosyl)-1,2,4-Oxadiazoles **9**

To a solution of the corresponding *O*-acyl-*C*-(2,3,4,6-tetra-*O*-benzoyl-β-D-glucopyranosyl)formamidoxime (**8**) in anhydrous toluene (3 mL/100 mg substrate), a catalytic amount of tetrabutylammonium fluoride (0.1 equiv., 1 M solution in THF) was added. The reaction mixture was heated at boiling temperature until TLC (EtOAc-hexane 1:2) showed that the starting material was completely consumed. When the reaction was complete the solvent was removed under reduced pressure and the resulting crude product was purified by column chromatography. 

#### 3.1.4. General Procedure 3 for the Synthesis of 5-(4-Substituted-Benzyl)-2-(2,3,4,6-Tetra-*O*-Benzoyl-β-D-Glucopyranosyl)-1,3,4-Oxadiazoles **12**

The 5-(2,3,4,6-tetra-*O*-benzoyl-β-D-glucopyranosyl)tetrazole (**11**) and the corresponding acid chloride (3 equiv.) were suspended in anhydrous *m*-xylene (3 mL/100 mg tetrazole) and the reaction mixture was heated at 140 °C. After completion of the reaction monitored by TLC (EtOAc-hexane 2:3), the mixture was evaporated under reduced pressure and the resulting crude product was purified by column chromatography. 

#### 3.1.5. General Procedure 4 for Cleavage of the *O*-Benzoyl Protecting Groups by the Zemplén Method to Get Test Compounds **10** and **13**

An *O*-benzoyl protected compound was dissolved in an anhydrous MeOH-CHCl_3_ solvent mixture (5 mL:1 mL/100 mg substrate) and a catalytic amount of ~1 M solution of NaOMe in MeOH was added. The reaction mixture was left to stand at rt until the TLC (CHCl_3_–MeOH 7:3) indicated complete transformation. The reaction mixture was then neutralized with Amberlyst 15 (hydrogen form), filtered, and the solvent was removed in vacuo. The crude product was purified by column chromatography.

#### 3.1.6. General Procedure 5 for the Synthesis of 4-Substituted-2-(2,3,4,6-Tetra-*O*-Benzyl-β-D-Glucopyranosyl)Pyrimidines **15**

To a solution of *C*-(β-D-glucopyranosyl)formamidine hydrochloride (**14**) in anhydrous CH_3_CN (2 mL/100 mg substrate,) the corresponding 4-(trimethylsilyl)but-3-in-2-one (1 equiv.), Na_2_CO_3_ (2 equiv.), and one drop of water were added. The reaction mixture was heated under reflux temperature for 8 h, then treated with activated charcoal and filtered. The solution was evaporated in vacuo and the residue was purified by column chromatography.

#### 3.1.7. General Procedure 6 for Removal of *O*-Benzyl Protecting Groups by Using BCl_3_ to Get Test Compounds **16** and **18**

The corresponding *O*-perbenzylated *C*-glucosyl heterocycle (**15** or **17**) was dissolved in anhydrous CH_2_Cl_2_ (5 mL/100 mg substrate). The stirred reaction mixture was cooled to ‒78 °C and BCl_3_ (5 equiv., ~1 M solution in CH_2_Cl_2_) was added. The stirring was continued at this temperature and the reaction was monitored by TLC (EtOAc-hexane 1:2 and CHCl_3_–MeOH 3:1). After the complete disappearance of the starting material (4 h), MeOH (5 mL) was added to the reaction mixture and the mixture was warmed to rt. Solvents were removed under diminished pressure and the residue was purified by column chromatography (CHCl_3_–MeOH = 19:1 → 9:1 gradient).

#### 3.1.8. Synthesis and Characterization of the Compounds

O*-Phenylacetyl-*C*-(2,3,4,6-tetra-*O*-benzoyl-β-D-glucopyranosyl)formamidoxime* (**8a**). Prepared from amidoxime **7** (0.50 g, 0.80 mmol) and phenylacetyl chloride (116 µL, 0.88 mmol) according to genereal procedure 1. The reaction time was 1 d. Purified by column chromatography (EtOAc-hexane 1:2) to give 0.35 g (58%) white solid. R_f_ = 0.58 (EtOAc-toluene 1:2); mp: 161–162 °C; [α]_D_ = +29 (c 0.27, CH_2_Cl_2_); ^1^H-NMR (360 MHz, CDCl_3_) δ (ppm): 8.03, 7.92, 7.90, 7.82 (4 × 2H, 4 d, *J* = 7.8 Hz in each, aromatics), 7.57–7.12 (17H, m, aromatics), 5.95, 5.74, 5.67 (3 × 1H, 3 pt, *J* = 9.8, 9.7 Hz in each, H-2′, H-3′, H-4′), 5.04 (2H, s, NH_2_), 4.63 (1H, dd, *J* = 12.4, 2.1 Hz, H-6′a), 4.49 (1H, dd, *J* = 12.4, 5.3 Hz, H-6′b), 4.46 (1H, d, *J* = 9.7 Hz, H-1′), 4.23 (1H, ddd, *J* = 9.8, 5.3, 2.1 Hz, H-5′), 3.60, 3.53 (2 × 1H, 2 d, *J* = 15.4 Hz in each, CH_2_); ^13^C-NMR (90 MHz, CDCl_3_) δ (ppm): 168.2, 166.1, 165.5, 165.5, 165.2 (C=O), 153.5 (C=N), 133.5–127.0 (aromatics), 76.6, 75.5, 73.5, 69.8, 69.0 (C-1′–C-5′), 62.9 (C-6′), 39.8 (*C*H_2_). ESI-HRMS positive mode (*m/z*): calcd. for C_43_H_36_N_2_NaO_11_^+^ [M + Na]^+^ 779.2211. Found: 779.2215.

O*-(4-Methoxyphenylacetyl)-*C*-(2,3,4,6-tetra-*O*-benzoyl-β-D-glucopyranosyl)formamidoxime* (**8b**). Prepared from amidoxime **7** (0.70 g, 1.1 mmol) and 4-methyoxyphenylacetyl chloride (186 µL, 1.2 mmol) according to general procedure 1. The reaction time was 1 d. Purified by column chromatography (EtOAc-hexane 1:2) to give 0.49 g (57%) white solid. R_f_ = 0.53 (EtOAc-toluene 1:2); mp: 162–164 °C; [α]_D_ = +55 (c 0.23, CH_2_Cl_2_); ^1^H-NMR (360 MHz, CDCl_3_) δ (ppm): 8.03, 7.92, 7.90, 7.82 (4 × 2H, 4 d, *J* = 7.4 Hz in each, aromatics), 7.57–7.21 (12H, m, aromatics), 7.05, 6.78 (2 × 1H, 2 d, *J* = 8.5 Hz in each, aromatics), 5.95, 5.74, 5.66 (3 × 1H, 3 pt, *J* = 9.8, 9.7 Hz in each, H-2′, H-3′, H-4′), 5.05 (2H, s, NH_2_), 4.63 (1H, dd, *J* = 12.5, 2.5 Hz, H-6′a), 4.49 (1H, dd, *J* = 12.5, 5.3 Hz, H-6′b), 4.46 (1H, d, *J* = 9.8 Hz, H-1′), 4.23 (1H, ddd, *J* = 9.8, 5.3, 2.5 Hz, H-5′), 3.75 (3H, s, CH_3_), 3.54, 3.47 (2 × 1H, 2 d, *J* = 15.5 Hz in each, CH_2_); ^13^C-NMR (90 MHz, CDCl_3_) δ (ppm): 168.6, 166.1, 165.5, 165.5, 165.2 (C=O), 153.4 (C=N), 158.6, 133.5, 133.3, 133.2, 130.0–128.2, 125.5, 113.9 (aromatics), 76.6, 75.6, 73.5, 69.8, 69.0 (C-1′–C-5′), 62.9 (C-6′), 55.1 (O*C*H_3_), 38.9 (*C*H_2_). ESI-HRMS positive mode (*m/z*): calcd. for C_44_H_38_N_2_NaO_12_^+^ [M + Na]^+^ 809.2317. Found: 809.2321.

O*-(4-Chlorophenylacetyl)-*C*-(2,3,4,6-tetra-*O*-benzoyl-β-D-glucopyranosyl)formamidoxime* (**8c**). Prepared from amidoxime **7** (0.70 g, 1.1 mmol) and 4-chlorophenylacetyl chloride (178 µL, 1.2 mmol) according to general procedure 1. The reaction time was 1 d. Purified by column chromatography (EtOAc-hexane 1:2) to give 0.53 g (61%) white solid. R_f_ = 0.59 (EtOAc-toluene 1:2); mp: 162–165 °C; [α]_D_ = +48 (c 0.22, CH_2_Cl_2_); ^1^H-NMR (360 MHz, CDCl_3_) δ (ppm): 8.03, 7.92, 7.90, 7.82 (4 × 2H, 4 d, *J* = 7.8 Hz in each, aromatics), 7.58–7.02 (16H, m, aromatics), 5.96, 5.74, 5.66 (3 × 1H, 3 pt, *J* = 9.8, 9.7 Hz in each, H-2′, H-3′, H-4′), 5.09 (2H, s, NH_2_), 4.64 (1H, dd, *J* = 12.4, 2.5 Hz, H-6′a), 4.50 (1H, dd, *J* = 12.4, 5.3 Hz, H-6′b), 4.46 (1H, d, *J* = 9.8 Hz, H-1′), 4.24 (1H, ddd, *J* = 9.7, 5.3, 2.5 Hz, H-5′), 3.56, 3.48 (2 × 1H, 2 d, *J* = 15.6 Hz in each, CH_2_); ^13^C-NMR (90 MHz, CDCl_3_) δ (ppm): 168.1, 166.1, 165.5, 165.5, 165.2 (C=O), 153.5 (C=N), 133.6–128.3 (aromatics), 76.6, 75.5, 73.5, 69.9, 69.0 (C-1′–C-5′), 62.9 (C-6′), 39.0 (*C*H_2_). ESI-HRMS positive mode (*m/z*): calcd. for C_43_H_35_ClN_2_NaO_11_^+^ [M + Na]^+^ 813.1822. Found: 813.1829.

*5-Benzyl-3-(2,3,4,6-tetra-*O*-benzoyl-β-D-glucopyranosyl)-1,2,4-oxadiazole* (**9a**). Prepared from compound **8a** (0.25 g, 0.33 mmol) according to general procedure 2. The reaction time was 4 h. Purified by column chromatography (EtOAc-hexane 1:4) to give 0.17 g (70%) pale yellow syrup. R_f_ = 0.44 (EtOAc-hexane 1:2); [α]_D_ = ‒15 (c 0.21, CH_2_Cl_2_); ^1^H-NMR (360 MHz, CDCl_3_) δ (ppm): 8.00, 7.92, 7.84, 7.78 (4 × 2H, 4 d, *J* = 7.6 Hz in each, aromatics), 7.52–7.18 (17H, m, aromatics), 6.06, 6.03, 5.85 (3 × 1H, 3 pt, *J* = 9.4, 9.3 Hz in each, H-2′, H-3′, H-4′), 5.11 (1H, d, *J* = 9.3 Hz, H-1′), 4.66 (1H, dd, *J* = 12.4, 2.7 Hz, H-6′a), 4.55 (1H, dd, *J* = 12.4, 5.4 Hz, H-6′b), 4.35 (1H, ddd, *J* = 9.4, 5.4, 2.7 Hz, H-5′), 4.22, 4.16 (2 × 1H, 2 d, *J* = 15.7 Hz in each, CH_2_); ^13^C-NMR (90 MHz, CDCl_3_) δ (ppm): 178.9 (C-5), 166.3, 166.0, 165.7, 165.0, 164.5 (C=O, C-3), 133.4, 133.2, 133.2, 133.0, 132.9, 129.7–127.4 (aromatics), 76.9, 74.0, 72.3, 70.5, 69.3 (C-1′–C-5′), 63.2 (C-6′), 32.8 (*C*H_2_). ESI-HRMS positive mode (*m/z*): calcd. for C_43_H_34_N_2_NaO_10_^+^ [M + Na]^+^ 761.2106. Found: 761.2107.

*5-(4-Methoxybenzyl)-3-(2,3,4,6-tetra-*O*-benzoyl-β-D-glucopyranosyl)-1,2,4-oxadiazole* (**9b**). Prepared from compound **8b** (0.36 g, 0.45 mmol) according to general procedure 2. The reaction time was 4 h. Purified by column chromatography (EtOAc-hexane 1:4) to give 0.31 g (91%) pale yellow syrup. R_f_ = 0.77 (EtOAc-hexane 1:1); [α]_D_ = ‒43 (c 0.21, CH_2_Cl_2_); ^1^H-NMR (360 MHz, CDCl_3_) δ (ppm): 8.00, 7.92, 7.84, 7.79 (4 × 2H, 4 d, *J* = 7.5 Hz in each, aromatics), 7.52–7.23 (12H, m, aromatics), 7.11, 6.75 (2 × 2H, 2 d, *J* = 8.5 Hz in each, aromatics), 6.05, 6.02, 5.84 (3 × 1H, 3 pt, *J* = 9.5, 9.3 Hz in each, H-2′, H-3′, H-4′), 5.10 (1H, d, *J* = 9.3 Hz, H-1′), 4.65 (1H, dd, *J* = 12.3, 2.8 Hz, H-6′a), 4.54 (1H, dd, *J* = 12.3, 5.2 Hz, H-6′b), 4.34 (1H, ddd, *J* = 9.5, 5.2, 2.8 Hz, H-5′), 4.16, 4.10 (2 × 1H, 2 d, *J* = 15.8 Hz in each, CH_2_), 3.72 (3H, s, CH_3_); ^13^C-NMR (90 MHz, CDCl_3_) δ (ppm): 179.2 (C-5), 166.3, 166.0, 165.7, 165.1, 164.5 (C=O, C-3), 158.9, 133.4, 133.2, 133.0, 129.8–128.2, 124.9, 114.1 (aromatics), 76.9, 74.0, 72.3, 70.5, 69.3 (C-1′–C-5′), 63.2 (C-6′), 55.1 (O*C*H_3_), 32.0 (*C*H_2_). ESI-HRMS positive mode (*m/z*): calcd. for C_44_H_36_N_2_NaO_11_^+^ [M + Na]^+^ 791.2211. Found: 791.2221.

*5-(4-Chlorobenzyl)-3-(2,3,4,6-tetra-*O*-benzoyl-β-D-glucopyranosyl)-1,2,4-oxadiazole* (**9c**). Prepared from compound **8c** (0.35 g, 0.44 mmol) according to general procedure 2. The reaction time was 4 h. Purified by column chromatography (EtOAc-hexane 1:4) to give 0.32 g (95%) colourless syrup. R_f_ = 0.43 (EtOAc-hexane 1:2); [α]_D_ = +52 (c 0.22, CH_2_Cl_2_); ^1^H-NMR (360 MHz, CDCl_3_) δ (ppm): 8.00, 7.92, 7.84, 7.77 (4 × 2H, 4 d, *J* = 7.6 Hz in each, aromatics), 7.52–7.10 (16H, m, aromatics), 6.06, 6.01, 5.85 (3 × 1H, 3 pt, *J* = 9.5, 9.4 Hz in each, H-2′, H-3′, H-4′), 5.11 (1H, d, *J* = 9.4 Hz, H-1′), 4.66 (1H, dd, *J* = 12.4, 2.7 Hz, H-6′a), 4.55 (1H, dd, *J* = 12.4, 5.2 Hz, H-6′b), 4.37 (1H, ddd, *J* = 9.5, 5.2, 2.7 Hz, H-5′), 4.17, 4.11 (2 × 1H, 2 d, *J* = 15.8 Hz in each, CH_2_); ^13^C-NMR (90 MHz, CDCl_3_) δ (ppm): 178.4 (C-5), 166.4, 166.0, 165.7, 165.0, 164.5 (C=O, C-3), 133.4, 133.4, 133.3, 133.2, 133.0, 131.3, 130.0–128.2 (aromatics), 76.9, 73.9, 72.3, 70.5, 69.3 (C-1′–C-5′), 63.2 (C-6′), 32.1 (*C*H_2_). ESI-HRMS positive mode (*m/z*): calcd. for C_43_H_33_ClN_2_NaO_10_^+^ [M + Na]^+^ 795.1716. Found: 795.1719.

*5-Benzyl-3-(β-D-glucopyranosyl)-1,2,4-oxadiazole* (**10a**). Prepared from compound **9a** (0.14 g, 0.19 mmol) according to general procedure 4. Purified by column chromatography (CHCl_3_–MeOH 8:1) to give 56 mg (92%) colourless syrup. R_f_ = 0.53 (CHCl_3_–MeOH 4:1); [α]_D_ = ‒63 (c 0.21, MeOH); ^1^H-NMR (360 MHz, CD_3_OD) δ (ppm): 7.35–7.26 (5H, m, Ph), 4.42 (1H, d, *J* = 9.7 Hz, H-1′), 4.30 (2H, s, CH_2_), 3.85 (1H, dd, *J* = 12.0, <1 Hz, H-6′a), 3.72–3.64, 3.51–3.41 (5H, m, H-2′, H-3′, H-4′, H-5′, H-6′b); ^13^C-NMR (90 MHz, CD_3_OD) δ (ppm): 180.5 (C-5), 169.3 (C-3), 135.0, 130.1 (2), 130.0 (2), 128.7 (Ph), 82.6, 79.3, 74.9, 73.3, 71.3 (C-1′–C-5′), 62.8 (C-6′), 33.6 (*C*H_2_). ESI-HRMS positive mode (*m/z*): calcd. for C_15_H_18_N_2_NaO_6_^+^ [M + Na]^+^ 345.1057. Found: 345.1057.

*3-(β-D-Glucopyranosyl)-5-(4-methoxybenzyl)-1,2,4-oxadiazole* (**10b**). Prepared from compound **9b** (0.22 g, 0.29 mmol) according to general procedure 4. Purified by column chromatography (CHCl_3_–MeOH 8:1) to give 80 mg (79%) colourless syrup. R_f_ = 0.53 (CHCl_3_–MeOH 4:1); [α]_D_ = ‒16 (c 0.22, MeOH); ^1^H-NMR (360 MHz, CD_3_OD) δ (ppm): 7.25, 6.89 (2 × 2H, 2 d, *J* = 8.5 Hz in each, aromatics), 4.42 (1H, d, *J* = 9.7 Hz, H-1′), 4.22 (2H, s, CH_2_), 3.85 (1H, dd, *J* = 12.0, < 1 Hz, H-6′a), 3.77 (3H, s, OCH_3_), 3.72–3.65, 3.51–3.42 (5H, m, H-2′, H-3′, H-4′, H-5′, H-6′b); ^13^C-NMR (90 MHz, CD_3_OD) δ (ppm): 180.9 (C-5), 169.3 (C-3), 160.6, 131.2 (2), 126.9, 115.3 (2) (aromatics), 82.6, 79.3, 74.9, 73.3, 71.3 (C-1′–C-5′), 62.8 (C-6′), 55.8 (O*C*H_3_), 32.7 (*C*H_2_). ESI-HRMS positive mode (*m/z*): calcd. for C_16_H_20_N_2_NaO_7_^+^ [M+Na]^+^ 375.1163. Found: 375.1162.

*5-(4-Chlorobenzyl)-3-(β-D-glucopyranosyl)-1,2,4-oxadiazole* (**10c**). Prepared from compound **9c** (0.32 g, 0.41 mmol) according to general procedure 4. Purified by column chromatography (CHCl_3_–MeOH 9:1) to give 115 mg (78%) colourless syrup. R_f_ = 0.53 (CHCl_3_–MeOH 4:1); [α]_D_ = ‒63 (c 0.21, MeOH); ^1^H-NMR (360 MHz, CD_3_OD) δ (ppm): 7.34 (4H, s, aromatics), 4.44 (1H, d, *J* = 9.7 Hz, H-1′), 4.30 (2H, s, CH_2_), 3.85 (1H, dd, *J* = 12.0, < 1 Hz, H-6′a), 3.73–3.65, 3.52–3.43 (5H, m, H-2′, H-3′, H-4′, H-5′, H-6′b); ^13^C-NMR (90 MHz, CD_3_OD) δ (ppm): 180.1 (C-5), 169.4 (C-3), 134.5, 133.7, 131.8 (2), 130.0 (2) (aromatics), 82.6, 79.2, 74.8, 73.3, 71.2 (C-1′–C-5′), 62.8 (C-6′), 32.8 (*C*H_2_). ESI-HRMS positive mode (*m/z*): calcd. for C_15_H_17_ClN_2_NaO_6_^+^ [M + Na]^+^ 379.0667. Found: 379.0667.

*5-Benzyl-2-(2,3,4,6-tetra-*O*-benzoyl-β-D-glucopyranosyl)-1,3,4-oxadiazole* (**12a**). Prepared from tetrazole **11** (0.10 g, 0.15 mmol) and phenylacetyl chloride (61 µL, 0.46 mmol) according to general procedure 3. The reaction time was 5 h. Purified by column chromatography (EtOAc-hexane 1:3) to give 70 mg (61%) pale yellow amorphous solid. R_f_ = 0.44 (EtOAc-hexane 2:3); [α]_D_ = +91 (c 0.11, CH_2_Cl_2_); ^1^H-NMR (360 MHz, CDCl_3_) δ (ppm): 8.01, 7.91, 7.82, 7.76 (4 × 2H, 4 d, *J* = 7.6 Hz in each, aromatics), 7.56–7.25 (17H, m, aromatics), 6.04, 5.85, 5.80 (3 × 1H, 3 pt, *J* = 9.8, 9.6 Hz in each, H-2′, H-3′, H-4′), 5.16 (1H, d, *J* = 9.8 Hz, H-1′), 4.64 (1H, dd, *J* = 12.4, 2.5 Hz, H-6′a), 4.49 (1H, dd, *J* = 12.4, 5.2 Hz, H-6′b), 4.31 (1H, ddd, *J* = 9.8, 5.2, 2.5 Hz, H-5′), 4.24, 4.16 (2 × 1H, 2 d, *J* = 15.9 Hz in each, CH_2_); ^13^C-NMR (90 MHz, CDCl_3_) δ (ppm): 166.8, 166.0, 165.6, 165.1, 164.6, 161.6 (C=O, C-2, C-5), 133.5, 133.4, 133.3, 133.1, 129.8–127.4 (aromatics), 77.0, 73.5, 71.8, 70.2, 69.0 (C-1′–C-5′), 62.9 (C-6′), 31.7 (*C*H_2_). ESI-HRMS positive mode (*m/z*): calcd. for C_43_H_34_N_2_NaO_10_^+^ [M + Na]^+^ 761.2106. Found: 761.2108.

*5-(4-Methoxybenzyl)-2-(2,3,4,6-tetra-*O*-benzoyl-β-D-glucopyranosyl)-1,3,4-oxadiazole* (**12b**). Prepared from tetrazole **11** (0.10 g, 0.15 mmol) and 4-methoxyphenylacetyl chloride (71 µL, 0.46 mmol) according to general procedure 3. The reaction time was 4 h. Purified by column chromatography (EtOAc-hexane 1:3) to give 83 mg (70%) pale yellow amorphous solid. R_f_ = 0.28 (EtOAc-hexane 2:3); [α]_D_ = +15 (c 0.21, CH_2_Cl_2_); ^1^H-NMR (360 MHz, CDCl_3_) δ (ppm): 8.01, 7.91, 7.82, 7.76 (4 × 2H, 4 d, *J* = 7.3 Hz in each, aromatics), 7.56–7.25 (12H, m, aromatics), 7.16, 6.80 (2 × 2H, 2 d, *J* = 8.6 Hz in each, aromatics), 6.04, 5.85, 5.80 (3 × 1H, 3 pt, *J* = 9.9, 9.7 Hz in each, H-2′, H-3′, H-4′), 5.16 (1H, d, *J* = 9.9 Hz, H-1′), 4.64 (1H, dd, *J* = 12.4, 2.6 Hz, H-6′a), 4.49 (1H, dd, *J* = 12.4, 5.2 Hz, H-6′b), 4.31 (1H, ddd, *J* = 9.7, 5.2, 2.6 Hz, H-5′), 4.17, 4.10 (2 × 1H, 2 d, *J* = 16.0 Hz in each, CH_2_), 4.08 (3H, s, OCH_3_); ^13^C-NMR (90 MHz, CDCl_3_) δ (ppm): 167.1, 166.0, 165.6, 165.1, 164.6, 161.5 (C=O, C-2, C-5), 158.8, 133.5, 133.4, 133.3, 133.1, 130.3–125.2, 114.2, 114.0 (aromatics), 77.0, 73.5, 71.7, 70.2, 69.0 (C-1′–C-5′), 62.9 (C-6′), 55.1 (O*C*H_3_), 30.8 (*C*H_2_). ESI-HRMS positive mode (*m/z*): calcd. for C_44_H_36_N_2_NaO_11_^+^ [M + Na]^+^ 791.2211. Found: 791.2217.

*5-(4-Chlorobenzyl)-2-(2,3,4,6-tetra-*O*-benzoyl-β-D-glucopyranosyl)-1,3,4-oxadiazole* (**12c**). Prepared from tetrazole **11** (0.50 g, 0.77 mmol) and 4-chlorophenylacetyl chloride (0.34 mL, 2.3 mmol) according to general procedure 3. The reaction time was 2 h. Purified by column chromatography (EtOAc-hexane 1:3) to give 0.36 g (61%) white amorphous solid. R_f_ = 0.33 (EtOAc-hexane 2:3); [α]_D_ = +38 (c 0.21, CH_2_Cl_2_); ^1^H-NMR (360 MHz, CDCl_3_) δ (ppm): 8.01, 7.91, 7.82, 7.75 (4 × 2H, 4 d, *J* = 7.5 Hz in each, aromatics), 7.57–7.16 (16H, m, aromatics), 6.05, 5.83, 5.81 (3 × 1H, 3 pt, *J* = 9.7, 9.6 Hz in each, H-2′, H-3′, H-4′), 5.17 (1H, d, *J* = 9.7 Hz, H-1′), 4.65 (1H, dd, *J* = 12.4, 2.2 Hz, H-6′a), 4.49 (1H, dd, *J* = 12.4, 5.2 Hz, H-6′b), 4.33 (1H, ddd, *J* = 9.7, 5.2, 2.2 Hz, H-5′), 4.21, 4.12 (2 × 1H, 2 d, *J* = 16.0 Hz in each, CH_2_); ^13^C-NMR (90 MHz, CDCl_3_) δ (ppm): 166.3, 166.0, 165.6, 165.1, 164.7, 161.7 (C=O, C-2, C-5), 133.5, 133.5, 133.3, 133.2, 131.7, 130.0–128.1 (aromatics), 77.0, 73.4, 71.7, 70.2, 68.9 (C-1′–C-5′), 62.9 (C-6′), 31.0 (*C*H_2_). ESI-HRMS positive mode (*m/z*): calcd. for C_43_H_33_ClN_2_NaO_10_^+^ [M + Na]^+^ 795.1716. Found: 795.1711.

*5-Benzyl-2-(β-D-glucopyranosyl)-1,3,4-oxadiazole* (**13a**). Prepared from compound **12a** (105 mg, 0.14 mmol) according to general procedure 4. Purified by column chromatography (CHCl_3_–MeOH 9:1) to give 32 mg (70%) colourless syrup. R_f_ = 0.43 (CHCl_3_–MeOH 4:1); [α]_D_ = ‒22 (c 0.17, MeOH); ^1^H-NMR (360 MHz, CD_3_OD) δ (ppm): 7.37–7.26 (5H, m, Ph), 4.52 (1H, d, *J* = 9.9 Hz, H-1′), 4.26 (2H, s, CH_2_), 3.86 (1H, dd, *J* = 12.2, 1.9 Hz, H-6′a), 3.69 (1H, pt, *J* = 9.9, 9.8 Hz, H-2′ or H-3′ or H-4′), 3.65 (1H, dd, *J* = 12.2, 5.4 Hz, H-6′b), 3.49–3.35 (3H, m, H-2′and / or H-3′and / or H-4′, H-5′); ^13^C-NMR (90 MHz, CD_3_OD) δ (ppm): 168.3, 166.1 (C-2, C-5), 135.2, 130.0 (4), 128.6 (Ph), 82.9, 79.2, 74.5, 73.4, 71.3 (C-1′–C-5′), 62.8 (C-6′), 32.3 (*C*H_2_). ESI-HRMS positive mode (*m/z*): calcd. for C_15_H_18_N_2_NaO_6_^+^ [M + Na]^+^ 345.1057. Found: 345.1056.

*2-(β-D-Glucopyranosyl)-5-(4-methoxybenzyl)-1,3,4-oxadiazole* (**13b**). Prepared from compound **12b** (0.15 g, 0.20 mmol) according to general procedure 4. Purified by column chromatography (CHCl_3_–MeOH 9:1) to give 49 mg (71%) colourless syrup. R_f_ = 0.44 (CHCl_3_–MeOH 4:1); [α]_D_ = ‒91 (c 0.18, MeOH); ^1^H-NMR (360 MHz, CD_3_OD) δ (ppm): 7.24, 6.89 (2 × 2H, 2 d, *J* = 8.7 Hz in each, aromatics), 4.51 (1H, d, *J* = 9.9 Hz, H-1′), 4.18 (2H, s, CH_2_), 3.85 (1H, dd, *J* = 12.2, 1.9 Hz, H-6′a), 3.77 (3H, s, OCH_3_), 3.68 (1H, pt, *J* = 9.8, 9.8 Hz, H-2′ or H-3′ or H-4′), 3.65 (1H, dd, *J* = 12.2, 5.4 Hz, H-6′b), 3.49–3.35 (3H, m, H-2′and / or H-3′and / or H-4′, H-5′); ^13^C-NMR (90 MHz, CD_3_OD) δ (ppm): 168.7, 166.1 (C-2, C-5), 160.7, 131.1 (2), 127.0, 115.4 (2) (aromatics), 82.9, 79.2, 74.5, 73.4, 71.3 (C-1′–C-5′), 62.8 (C-6′), 55.8 (O*C*H_3_), 31.5 (*C*H_2_). ESI-HRMS positive mode (*m/z*): calcd. for C_16_H_20_N_2_NaO_7_^+^ [M + Na]^+^ 375.1163. Found: 375.1165.

*5-(4-Chlorobenzyl)-2-(β-D-glucopyranosyl)-1,3,4-oxadiazole* (**13c**). Prepared from compound **12c** (0.15 g, 0.19 mmol) according to general procedure 4. Purified by column chromatography (CHCl_3_–MeOH 9:1) to give 57 mg (83%) colourless syrup. R_f_ = 0.45 (CHCl_3_–MeOH 4:1); [α]_D_ = ‒17 (c 0.18, MeOH); ^1^H-NMR (360 MHz, CD_3_OD) δ (ppm): 7.37–7.31 (4H, m, aromatics), 4.53 (1H, d, *J* = 9.9 Hz, H-1′), 4.26 (2H, s, CH_2_), 3.85 (1H, dd, *J* = 12.2, 1.9 Hz, H-6′a), 3.69 (1H, pt, *J* = 9.8, 9.6 Hz, H-2′ or H-3′ or H-4′), 3.65 (1H, dd, *J* = 12.2, 5.4 Hz, H-6′b), 3.51–3.36 (3H, m, H-2′and / or H-3′and / or H-4′, H-5′); ^13^C-NMR (90 MHz, CD_3_OD) δ (ppm): 167.9, 166.2 (C-2, C-5), 134.6, 134.0, 131.7 (2), 130.0 (2) (aromatics), 82.9, 79.1, 74.5, 73.4, 71.3 (C-1′–C-5′), 62.7 (C-6′), 31.6 (*C*H_2_). ESI-HRMS positive mode (*m/z*): calcd. for C_15_H_17_ClN_2_NaO_6_^+^ [M + Na]^+^ 379.0667. Found: 379.0669.

*4-Benzyl-2-(2,3,4,6-tetra-*O*-benzyl-β-D-glucopyranosyl)pyrimidine* (**15a**). Prepared from amidine **14** (400 mg, 0.66 mmol), 1-phenyl-4-(trimethylsilyl)but-3-in-2-one (143 mg, 0.66 mmol) and Na_2_CO_3_ (141 mg, 1.33 mmol) according to general procedure 5. Purified by column chromatography (EtOAc-hexane 1:2) to give 308 mg (67%) pale yellow syrup. R_f_ = 0.29 (EtOAc-hexane 1:3); [α]_D_ = ‒116 (c 0.22, CH_2_Cl_2_); ^1^H-NMR (400 MHz, CDCl_3_) δ (ppm): 8.55 (1H, d, *J* = 5.1 Hz, H-6), 7.35–6.79 (25H, m, aromatics), 6.94 (1H, d, *J* = 5.1 Hz, H-5), 4.93 (2H, s, Ph*CH_2_*), 4.86, 4.58 (2 × 1H, 2d, *J* = 10.7 Hz, Ph*CH_2_*), 4.59, 4.11 (2 × 1H, 2d, *J* = 11.3 Hz, Ph*CH_2_*), 4.58 (1H, d, *J* = 9.7 Hz, H-1′), 4.58, 4.51 (2 × 1H, 2d, *J* = 12.2 Hz, Ph*CH_2_*), 4.20 (1H, pt, *J* = 9.7, 9.3 Hz, H-2′), 4.08 (2H, s, Ph*CH_2_*), 3.90 (1H, pt, *J* = 9.3, 9.2 Hz, H-3′ or H-4′), 3.81–3.70 (4H, m, H-3′ or H-4′, H-5′, H-6′a,b); ^13^C-NMR (100 MHz, CDCl_3_) δ (ppm): 169.8, 166.0 (C-2, C-4), 157.5 (C-6), 138.8, 138.3, 138.3, 138.2, 137.4, 129.4–127.0 (aromatics), 119.5 (C-5), 87.1, 83.3, 81.5, 79.0, 78.4 (C-1′–C-5′), 75.6, 75.2, 74.6, 73.5 (4 × Ph*C*H_2_), 69.2 (C-6′), 44.3 (Ph*C*H_2_). ESI-HRMS positive mode (*m/z*): calcd. for C_45_H_45_N_2_O_5_^+^ [M+H]^+^ 693.3323; C_45_H_44_N_2_NaO_5_^+^ [M+Na]^+^ 715.3142. Found: [M + H]^+^ 693.3322; [M+Na]^+^ 715.3141.

*4-(4-Methoxybenzyl)-2-(2,3,4,6-tetra-*O*-benzyl-β-D-glucopyranosyl)pyrimidine* (**15b**). Prepared from amidine **14** (400 mg, 0.66 mmol), 1-(4-methoxyphenyl)-4-(trimethylsilyl)but-3-in-2-one (163 mg, 0.66 mmol), and Na_2_CO_3_ (141 mg, 1.33 mmol) according to general procedure 5. Purified by column chromatography (EtOAc-hexane 1:2) to give 235 mg (49%) pale yellow syrup. R_f_ = 0.28 (EtOAc-hexane 1:2); [α]_D_ = ‒123 (c 0.22, CH_2_Cl_2_); ^1^H-NMR (360 MHz, CDCl_3_) δ (ppm): 8.56 (1H, d, *J* = 5.2 Hz, H-6), 7.35–6.77 (24H, m, aromatics), 6.94 (1H, d, *J* = 5.2 Hz, H-5), 4.93 (2H, s, Ph*CH_2_*), 4.86 (1H, d, *J* = 10.7 Hz, Ph*CH_2_*), 4.60–4.49 (5H, m, Ph*CH_2_*, H-1′), 4.20 (1H, pt, *J* = 9.5, 9.3 Hz, H-2′), 4.11 (1H, d, *J* = 11.2 Hz, Ph*CH_2_*), 4.02 (2H, s, *p*-OMe-C_6_H_4_*CH_2_*), 3.90 (1H, pt, *J* = 9.2, 9.1 Hz, H-3′ or H-4′), 3.82–3.67 (7H, m, H-3′ or H-4′, H-5′, H-6′a,b, OMe); ^13^C-NMR (90 MHz, CDCl_3_) δ (ppm): 170.3, 165.9 (C-2, C-4), 158.6 (C_q_, C_6_H_4_-OMe), 157.5 (C-6), 138.8, 138.3, 138.2, 138.1, 130.4, 129.5, 128.5–127.4, 114.3 (aromatics), 119.5 (C-5), 87.1, 83.2, 81.5, 79.9, 78.4 (C-1′–C-5′), 75.7, 75.2, 74.6, 73.5 (4 × Ph*C*H_2_), 69.2 (C-6′), 55.3 (O*C*H_3_), 43.4 (*p*-OMe-C_6_H_4_*C*H_2_). ESI-HRMS positive mode (*m/z*): calcd. for C_46_H_47_N_2_O_6_^+^ [M + H]^+^ 723.3429; C_46_H_46_N_2_NaO_6_^+^ [M+Na]^+^ 745.3248. Found: [M+H]^+^ 723.3434; [M + Na]^+^ 745.3255.

*4-(4-Chlorobenzyl)-2-(2,3,4,6-tetra-*O*-benzyl-β-D-glucopyranosyl)pyrimidine* (**15c**). Prepared from amidine **14** (500 mg, 0.83 mmol), 1-(4-chlorophenyl)-4-(trimethylsilyl)but-3-in-2-one (208 mg, 0.83 mmol), and Na_2_CO_3_ (176 mg, 1.66 mmol) according to general procedure 5. Purified by column chromatography (EtOAc-hexane 1:2) to give 280 mg (46%) pale yellow syrup. R_f_ = 0.25 (EtOAc-hexane 1:2); [α]_D_ = +67 (c 0.59, CH_2_Cl_2_); ^1^H-NMR (360 MHz, CDCl_3_) δ (ppm): 8.59 (1H, d, *J* = 5.0 Hz, H-6), 7.31–6.78 (24H, m, aromatics), 6.95 (1H, d, *J* = 5.0 Hz, H-5), 4.92 (2H, s, Ph*CH_2_*), 4.86 (1H, d, *J* = 10.7 Hz, Ph*CH_2_*), 4.59–4.49 (5H, m, Ph*CH_2_*, H-1′), 4.17 (1H, pt, *J* = 9.4, 9.3 Hz, H-2′ or H-3′ or H-4′), 4.08 (1H, d, *J* = 11.3 Hz, Ph*CH_2_*), 4.03 (2H, s, *p*-Cl-C_6_H_4_*CH_2_*), 3.90 (1H, pt, *J* = 9.1, 9.0 Hz, H-2′ or H-3′ or H-4′), 3.81–3.70 (4H, m, H-2′ or H-3′ or H-4′, H-5′, H-6′a,b); ^13^C-NMR (90 MHz, CDCl_3_) δ (ppm): 169.2, 166.2 (C-2, C-4), 157.7 (C-6), 138.8, 138.2, 138.2 (2), 135.9, 133.0, 130.7, 129.0–127.5 (aromatics), 119.5 (C-5), 87.1, 83.2, 81.5, 79.9, 78.4 (C-1′–C-5′), 75.7, 75.2, 74.6, 73.5 (4 × Ph*C*H_2_), 69.2 (C-6′), 43.5 (*p*-Cl-C_6_H_4_*C*H_2_). ESI-HRMS positive mode (*m/z*): calcd. for C_45_H_44_ClN_2_O_5_^+^ [M + H]^+^ 727.2933; C_45_H_43_ClN_2_NaO_5_^+^ [M + Na]^+^ 749.2753. Found: [M + H]^+^ 727.2943; [M + Na]^+^ 749.2763.

*4-(2-Naphthylmethyl)-2-(2,3,4,6-tetra-*O*-benzyl-β-D-glucopyranosyl)pyrimidine* (**15d**). Prepared from amidine **14** (400 mg, 0.66 mmol), 1-(2-naphthyl)-4-(trimethylsilyl)but-3-in-2-one (177 mg, 0.66 mmol), and Na_2_CO_3_ (141 mg, 1.33 mmol) according to general procedure 5. Purified by column chromatography (EtOAc-hexane 1:3) to give 281 mg (57%) pale yellow syrup. R_f_ = 0.28 (EtOAc-hexane 1:2); [α]_D_ = ‒71 (c 0.23, CH_2_Cl_2_); ^1^H-NMR (400 MHz, CDCl_3_) δ (ppm): 8.56 (1H, d, *J* = 5.1 Hz, H-6), 7.80–6.76 (27H, m, aromatics), 6.98 (1H, d, *J* = 5.1 Hz, H-5), 4.92 (2H, s, Ph*CH_2_*), 4.86 (1H, d, *J* = 10.8 Hz, Ph*CH_2_*), 4.60–4.50 (5H, m, Ph*CH_2_*, H-1′), 4.26 (2H, s, C_10_H_7_*CH_2_*), 4.20 (1H, pt, *J* = 9.5, 9.3 Hz, H-2′), 4.10 (1H, d, *J* = 11.2 Hz, Ph*CH_2_*), 3.90 (1H, pt, *J* = 9.2, 9.0 Hz, H-3′ or H-4′), 3.81–3.70 (4H, m, H-3′ or H-4′, H-5′, H-6′a,b); ^13^C-NMR (100 MHz, CDCl_3_) δ (ppm): 169.9, 166.1 (C-2, C-4), 157.6 (C-6), 138.8, 138.3, 138.2 (2), 134.9, 133.7, 132.5, 128.7–127.4, 126.4, 126.0 (aromatics), 119.7 (C-5), 87.1, 83.2, 81.5, 79.8, 78.3 (C-1′–C-5′), 75.7, 75.3, 74.7, 73.6 (4 × Ph*C*H_2_), 69.2 (C-6′), 44.4 (C_10_H_7_*C*H_2_). ESI-HRMS positive mode (*m/z*): calcd. for C_49_H_47_N_2_O_5_^+^ [M + H]^+^ 743.3479; C_49_H_46_N_2_NaO_5_^+^ [M + Na]^+^ 765.3299. Found: [M + H]^+^ 743.3490; [M + Na]^+^ 765.3310.

*4-Benzyl-2-(β-D-glucopyranosyl)pyrimidine* (**16a**). Method A: Prepared from compound **15a** (300 mg, 0.43 mmol) according to general procedure 6. Yield: 108 mg (75%) pale yellow syrup. Method B: To a solution of compound **15a** (200 mg, 0.29 mmol) in anhydrous CH_2_Cl_2_ (10 mL), EtSH (832 μL, 11.55 mmol) and BF_3_·Et_2_O (713 μL, 5.77 mmol) were added and the reaction mixture was stirred at rt. After completion of the reaction (3 d) judged by TLC (EtOAc-hexane 1:1 and CHCl_3_–MeOH 3:1), the reaction mixture was diluted with EtOAc (20 mL) and extracted with water (3 × 10 mL). The combined aqueous phase was evaporated in vacuo and the residue was purified by column chromatography (CHCl_3_–MeOH 19:1 → 9:1 gradient) to yield 82 mg (85%). R_f_ = 0.43 (CHCl_3_–MeOH 3:1); [α]_D_ = ‒77 (c 0.22, MeOH); ^1^H-NMR (360 MHz, CD_3_OD) δ (ppm): 8.66 (1H, d, *J* = 5.2 Hz, H-6), 7.34–7.23 (5H, m, Ph), 7.24 (1H, d, *J* = 5.2 Hz, H-5), 4.42 (1H, d, *J* = 9.6 Hz, H-1′), 4.16 (2H, s, CH_2_), 3.88 (1H, dd, *J* = 12.2, 1.8 Hz, H-6′a), 3.78 (1H, pt, *J* = 9.3, 9.0 Hz, H-2′ or H-3′ or H-4′), 3.72 (1H, dd, *J* = 12.2, 4.7 Hz, H-6′b), 3.58–3.45 (3H, m, H-2′ and/or H-3′ and/or H-4, H-5′); ^13^C-NMR (90 MHz, CD_3_OD) δ (ppm): 171.8, 167.7 (C-2, C-4), 158.5 (C-6), 138.8, 130.3 (2), 129.9 (2), 128.0 (Ph), 121.1 (C-5), 83.7, 82.5, 79.4, 74.8, 71.3 (C-1′–C-5′), 62.9 (C-6′), 44.5 (*C*H_2_). ESI-HRMS positive mode (*m/z*): calcd. for C_17_H_21_N_2_O_5_^+^ [M + H]^+^ 333.1445; C_17_H_20_N_2_NaO_5_^+^ [M + Na]^+^ 355.1264. Found: [M + H]^+^ 333.1447; [M + Na]^+^ 355.1266.

*2-(β-D-Glucopyranosyl)-4-(4-methoxybenzyl)pyrimidine* (**16b**). Prepared from compound **15b** (200 mg, 0.28 mmol) according to general procedure 6. Yield: 49 mg (49%), pale yellow syrup. R_f_ = 0.53 (CHCl_3_–MeOH 7:3). [α]_D_ = ‒60 (c 0.33, MeOH); ^1^H-NMR (400 MHz, CD_3_OD) δ (ppm): 8.63 (1H, d, *J* = 5.2 Hz, H-6), 7.20 (1H, d, *J* = 5.2 Hz, H-5), 7.19, 7.20 (2 × 2H, 2d, *J* = 8.7 Hz in each, aromatics), 4.42 (1H, d, *J* = 9.6 Hz, H-1′), 4.07 (2H, s, CH_2_), 3.88 (1H, dd, *J* = 12.2, 2.0 Hz, H-6′a), 3.76 (1H, pt, *J* = 9.6, 9.2 Hz, H-2′), 3.75 (3H, s, OCH_3_), 3.73 (1H, dd, *J* = 12.2, 4.9 Hz, H-6′b), 3.58 (1H, pt, *J* = 9.2, 9.0 Hz, H-3′ or H-4′) 3.51 (1H, pt, *J* = 9.4, 9.2 Hz, H-3′ or H-4′), 3.47 (1H, ddd, *J* = 9.4, 4.9, 2.0 Hz, H-5′); ^13^C-NMR (100 MHz, CD_3_OD) δ (ppm): 172.2, 167.5 (C-2, C-4), 160.1 (C_q_, C_6_H_4_-OMe), 158.4 (C-6), 131.3 (2), 130.6, 115.2 (2) (aromatics), 121.0 (C-5), 83.5, 82.4, 79.4, 74.7, 71.2 (C-1′–C-5′), 62.8 (C-6′), 55.7 (O*C*H_3_), 43.7 (*C*H_2_). ESI-HRMS positive mode (*m/z*): calcd. for C_18_H_23_N_2_O_6_^+^ [M + H]^+^ 363.1551; C_18_H_22_N_2_NaO_6_^+^ [M + Na]^+^ 385.1370. Found: [M + H]^+^ 363.1548; [M + Na]^+^ 385.1368.

*4-(4-Chlorobenzyl)-2-(β-D-glucopyranosyl)pyrimidine* (**16c**). Prepared from compound **15c** (280 mg, 0.39 mmol) according to general procedure 6. Yield: 110 mg (78%), pale yellow syrup. R_f_ = 0.56 (CHCl_3_–MeOH 3:1); [α]_D_ = ‒2 (c 0.31, MeOH); ^1^H-NMR (400 MHz, CD_3_OD) δ (ppm): 8.67 (1H, d, *J* = 5.2 Hz, H-6), 7.31–7.27 (4H, m, aromatics), 7.25 (1H, d, *J* = 5.2 Hz, H-5), 4.43 (1H, d, *J* = 9.6 Hz, H-1′), 4.13 (2H, s, CH_2_), 3.88 (1H, dd, *J* = 12.2, 1.9 Hz, H-6′a), 3.78 (1H, pt, *J* = 9.3, 9.2 Hz, H-2′ or H-3′ or H-4′), 3.73 (1H, dd, *J* = 12.2, 4.6 Hz, H-6′b), 3.63–3.56 (2H, m, H-2′ and/or H-3′ and/or H-4′), 3.52 (1H, pt, *J* = 9.3, 9.1 Hz, H-2′ or H-3′ or H-4′), 3.49–3.45 (1H, m, H-5′); ^13^C-NMR (90 MHz, CD_3_OD) δ (ppm): 171.1, 167.7 (C-2, C-4), 158.6 (C-6), 137.5, 133.8, 131.9 (2), 129.8 (2) (aromatics), 121.1 (C-5), 83.6, 82.4, 79.3, 74.7, 71.2 (C-1′–C-5′), 62.8 (C-6′), 43.7 (*C*H_2_). ESI-HRMS positive mode (*m/z*): calcd. for C_17_H_19_ClN_2_NaO_5_^+^ [M + Na]^+^ 389.0875. Found: 389.0875.

*2-(β-D-Glucopyranosyl)-4-(2-naphthylmethyl)pyrimidine* (**16d**). Prepared from compound **15d** (200 mg, 0.27 mmol) according to general procedure 6. Yield: 72 mg (70%), pale yellow syrup. R_f_ = 0.53 (CHCl_3_–MeOH 7:3); [α]_D_ = ‒92 (c 0.26, MeOH); ^1^H-NMR (400 MHz, CD_3_OD) δ (ppm): 8.61 (1H, d, *J* = 5.2 Hz, H-6), 7.79–7.34 (7H, m, aromatics), 7.20 (1H, d, *J* = 5.2 Hz, H-5), 4.45 (1H, d, *J* = 9.6 Hz, H-1′), 4.27 (2H, s, CH_2_), 3.89 (1H, dd, *J* = 12.2, 2.1 Hz, H-6′a), 3.80 (1H, pt, *J* = 9.6, 9.0 Hz, H-2′), 3.74 (1H, dd, *J* = 12.2, 4.8 Hz, H-6′b), 3.59 (1H, pt, *J* = 9.3, 9.0 Hz, H-3′ or H-4′), 3.53 (1H, pt, *J* = 9.3, 9.1 Hz, H-3′ or H-4′) 3.50–3.47 (1H, m, H-5′); ^13^C-NMR (100 MHz, CD_3_OD) δ (ppm): 171.6, 167.6 (C-2, C-4), 158.5 (C-6), 136.2, 135.0, 133.8, 129.5, 128.9, 128.6, 128.6, 128.3, 127.3, 126.8 (aromatics), 121.2 (C-5), 83.5, 82.3, 79.3, 74.7, 71.2 (C-1′–C-5′), 62.8 (C-6′), 44.6 (CH_2_). ESI-HRMS positive mode (*m/z*): calcd. for C_21_H_23_N_2_O_5_^+^ [M + H]^+^ 383.1601; C_21_H_22_N_2_NaO_5_^+^ [M + Na]^+^ 405.1421. Found: [M + H]^+^ 383.1602; [M + Na]^+^ 405.1421.

*4(5)-Benzyl-2-(2,3,4,6-tetra-*O*-benzyl-β-D-glucopyranosyl)imidazole* (**17**). *C*-(2,3,4,6-Tetra-*O*-benzyl-β-D-glucopyranosyl)formamidine hydrochloride (**14**, 0.20 g, 0.33 mmol) was dissolved in a THF-H_2_O solvent mixture (8 mL and 1 mL, respectively) and K_2_CO_3_ (0.09 g, 0.66 mmol, 2 equiv.) was added. The mixture was stirred at rt. After 15 min, 1-bromo-3-phenylpropan-2-one (71 mg, 0.33 mmol, 1 equiv.) was added to the reaction mixture and the stirring was continued at rt. The conversion of the starting material was judged by TLC (9:1 CHCl_3_–MeOH and 1:1 hexane-EtOAc). After two days, the mixture was diluted with EtOAc (20 mL) and extracted with water (2 × 10 mL). The organic phase was dried over MgSO_4_, filtered, and evaporated. The residue was purified by column chromatography (2:1 hexane-EtOAc) to give 89 mg (39%) pale yellow syrup. R_f_ = 0.51 (1:1 hexane-EtOAc); [α]_D_ = +2 (c 0.24, CH_2_Cl_2_);^1^H-NMR (360 MHz, CDCl_3_) δ (ppm): 9.92 (1H, br s, NH), 7.29–6.91 (26H, m, aromatics, imidazole CH), 4.92, 4.82 (2 × 1H, 2 d, *J* = 11.1 Hz in each, Ph*CH_2_*), 4.81, 4.49 (2 × 1H, 2 d, *J* = 10.8 Hz in each, Ph*CH_2_*), 4.44–4.29 (4H, m, 3 × Ph*CH_2_*, H-1′), 4.18 (1H, d, *J* = 10.5 Hz, Ph*CH_2_*), 3.88–3.83 (3H, m, Ph*CH_2_*, H-2′ or H-3′ or H-4′), 3.74 (1H, pt, *J* = 8.9, 8.8 Hz, H-2′ or H-3′ or H-4′), 3.64–3.50 (4H, m, H-2′ or H-3′ or H-4′, H-5′, H-6′a,b); ^13^C-NMR (90 MHz, CDCl_3_) δ (ppm): 144.4 (C-2), 138.6, 138.0, 137.9 (2), 137.6, 128.7–127.5 (aromatics, C-4), 126.1 (C-4), 86.3, 81.6, 78.9, 77.8, 75.1 (C-1′–C-5′), 75.5, 74.9, 74.6, 73.1 (4 × Ph*C*H_2_), 68.9 (C-6′). ESI-HRMS positive mode (*m/z*): calcd. for C_44_H_44_N_2_NaO_5_^+^ [M + Na]^+^ 703.3142. Found: 703.3141.

*4(5)-Benzyl-2-(β-D-glucopyranosyl)imidazole* (**18**). Prepared from compound **17** (148 mg, 0.22 mmol) according to general procedure 6. Purified by columnn chromatography (CHCl_3_–MeOH 8:1) to give 58 mg (83%) colourless syrup. R_f_ = 0.47 (CHCl_3_–MeOH 7:3); [α]_D_ = +14 (c 0.21, MeOH); ^1^H-NMR (360 MHz, CD_3_OD) δ (ppm): 7.28–7.15 (5H, m, aromatics), 6.70 (1H, s, imidazole CH), 4.30 (1H, d, *J* = 9.5 Hz, H-1′), 3.90 (2H, s, PhC*H_2_*), 3.85 (1H, dd, *J* = 12.0, < 1Hz, H-6′a), 3.69 (1H, dd, *J* = 12.0, 4.0 Hz, H-6′b), 3.59 (1H, pt, *J* = 9.1, 8.8 Hz, H-2′ or H-3′ or H-4′), 3.50–3.40 (3H, m, H-2′ and/or H-3′ and/or H-4′, H-5′); ^13^C-NMR (90 MHz, CD_3_OD) δ (ppm): 147.0 (C-2), 140.9 (Ph-C_q_), 137.4 (C-4), 129.7 (2), 129.5 (2), 127.3 (Ph), 119.1 (C-5), 82.0, 79.3, 76.7, 74.5, 71.3 (C-1′–C-5′), 62.8 (C-6′), 33.8 (Ph*C*H_2_). ESI-HRMS positive mode (*m/z*): calcd. for C_16_H_21_N_2_NO_5_^+^ [M + H]^+^ 321.1445. Found: 321.1445.

### 3.2. Biochemical Materials and Methods

#### 3.2.1. Reagents

The 2-NBDG (2-deoxy-2-[(7-nitro-2,1,3-benzoxadiazol-4-yl)amino]-D-glucose, cat. 11046) was purchased from Cayman Chemical Company (Ann Arbor, MI, USA). Lentivirus for SLC5A1 and SLC5A2 (NM_000343 for SGLT1, and NM-003041 for SGLT2) were obtained from Origene (Rockville, MD, USA). These expression vectors contain a puromycin resistance gene. Puromycin (1952692) was from ThermoFisher (Waltham, MA, USA), phlorizin (P3449–1G) and Triton-X (X-100X-100ML) were from Sigma-Aldrich (St. Luis, MO, USA), and dapagliflozin was from Carbosynth Ltd. (Compton, West Berkshire, UK). CyQUANT^TM^ Cell Proliferation Assay Kit (2036547) was from Invitrogen (Carlsbad, CA, USA). Glycogen phosphorylase *b* was isolated from rabbit skeletal muscle according to the method of Fischer and Krebs [78].

#### 3.2.2. Cell Culture

TSA201 (Human Embryonal Kidney) cells were grown in high glucose Dulbecco’s Modified Eagle’s Medium (DMEM, M8042, Sigma-Aldrich, St. Luis, MO, USA) in the presence of 10% Fetal Bovine Serum (FBS, F9665, Sigma-Aldrich and 1.5% L-glutamine (G7513-100ML, Sigma-Aldrich, St. Luis, MO, USA). The media of overexpressed cell lines contained 10 μg/mL puromycin. Cells were maintained in T-75 culture flasks at 37 °C in 5% CO_2_ atmosphere.

#### 3.2.3. Lentiviral Transfection

Cells (100,000 cells, TSA201) were plated into each well of a 24-well plate and incubated overnight at 37 °C. Six wells were transfected with lentiviral particles (NM_000343 for SGLT1, and NM-003041 for SGLT2) containing the mammalian expression vectors with SGLT1 or SGLT2 and the puromycin resistance gene, using 8 µg/mL hexadimetrine bromide (MOI = 3.5 for the best transfection). Two wells were treated only with hexadimetrine bromide as a control. After 24 h of transfection, the medium was changed to fresh medium without antibiotics. Cells were collected and transferred into T-25 flasks after 3 days. On the fifth day, 10 µg/mL puromycin was added to select only the transfected cells. The nontransfected cells cannot survive this high concentration of antibiotic. To keep up the selection, the medium including 10 µg/mL puromycin was replaced every two days. The cells lived under puromycin pressure until the day of the experiments. 

#### 3.2.4. Western Blot

Cells were grown in 60-mm petri dishes. Cells were washed with PBS (0.01 M phosphate, 0.0027 M KCl, 0.14 M NaCl, pH 7.4), then 0.1 mL of TET lysis Buffer (0.02 M Tris, 0.005 M EDTA, 1% Triton-X, pH= 7.4) was added to cells on ice and cells were collected. The lysate was incubated for 45 min on ice and vortexed 3 times. Proteins were separated on a gradient SDS-PAGE (Mini-PROTEAN TGX Gels, BIO-RAD, Hercules, CA, USA ) and the proteins were transferred to a PVDF membrane (88518, Thermo Fisher, Waltham, MA, USA ). The membrane was blocked with 5% Bovine Serum Albumin (BSA, A9647-100G, Sigma-Aldrich, St. Luis, MO, USA). The membrane was incubated with anti-SGLT1 (anti-SLC5A1 TA324226 from Origene, Rockville, MD, USA), anti-SGLT2 (ab137207, Abcam, Cambridge, UK), or anti-GAPDH (ABS16, EMD Millipore, Rockville, MD, USA) in 1% BSA solution. Super Signal West Femto and Pico Maximum Sensitivity Substrates (Sigma-Aldrich, St. Luis, MO, USA) were used for chemiluminescence signals. The results were quantitated using the ChemiDoc Touch Imaging System (Bio-Rad, Hercules, CA, USA). The data were analysed using Image Lab software from Bio-Rad (Hercules, CA, USA).

#### 3.2.5. Cytotoxicity Assay

TSA201 (Human Embryonal Kidney) cells were used for cytotoxicity assay. TSA201 were maintained in Dulbecco’s modified Eagle’s medium (DMEM, Sigma, St. Luis, MO, USA) containing 10% heat-inactivated fetal bovine serum, and 1.5% L-glutamine at 37 °C in humidified atmosphere of 5% CO_2_.

The cytotoxicities of all earlier and newly synthesized inhibitors were measured by fluorimetric CyQUANT assay. Cells (25,000/well) were plated into 96-well plates. The next day, the media was removed, and replaced with DMEM containing 10% FBS, 1.5% L-glutamine, and synthesized the compounds (1000 μM). Controls were treated with vehicle (DMSO). After 4 h, the supernatant was removed and cells were washed with PBS (Phosphate Buffer Solution pH 7.4). Then, the plates were frozen. The next day the plates were thawed and 200 mL CyQUANT reagent was added to each well. The plates were incubated for 5 min at room temperature, protected from light. The fluorescent signal was measured using a fluorimeter (excitation wavelength: 480 nm, emission wavelength: 520 nm). The obtained values were plotted against control. 

#### 3.2.6. 2-NBDG Uptake Measurement

TSA201 cells (25,000) with and without overexpressed SGLT1 or 2 were put into poly-L-lysine (P5999, Sigma-Aldrich, St. Luis, MO, USA) treated wells of a 96-well plate. The next day, the cells were starved for 1 h with 90 µL glucose-free medium (D5036-10X1L Sigma-Aldrich, St. Luis, MO, USA) (with or without inhibitor). After starving, cells were treated with 100 µM 2-NBDG and incubated for 30 min (again with or without inhibitor). The incubation time of cells with possible inhibitors was 90 min altogether. After incubation, the medium was removed, the wells were washed with PBS twice, lysed with 100 µL lysis buffer (0.1 M KH_2_PO_4_ + 1% Triton-x, pH = 11), and stored at -20°C. After thawing, the cells were resuspended and the fluorescent signal was measured using a fluorimeter (excitation wavelength: 485 nm, emission wavelength: 538 nm). Subsequently, the protein concentration was measured and NBDG uptake was normalized to protein amount.

#### 3.2.7. Calculation of IC_50_ for SGLT1 and 2

We selected the optimal concentration to identify the IC_50_ of the compounds using dose-response measurements, then we chose the optimal concentrations between 20–80% of inhibition. After selecting optimal concentrations, each compound was tested at four different concentrations. Concentrations of 0.1, 1, 10, 100, and 1000 μM were used and the lowest and highest concentrations were removed, depended on the dose-response measurements. Examples of dose-response curves for dapagliflozin and compounds **22b** and **24b** are shown in the Supplementary Figure S3. To determine the specific effects of the compounds on SGLT1 or 2, the glucose uptake values in TSA201 cells without SGLT overexpression were subtracted from the values in TSA201 cell overexpressing SGLT. Overexpressed control minus original control was 100%. All calculations were prepared using the GraphPad Prism software (GraphPad Software, Inc., SanDiego, CA, USA). The data were plotted on a logarithmic scale as a function of inhibitor concentration. The IC_50_ values were calculated by non-linear regression analysis from sigmoidal dose-response curves. The results of kinetic experiments were calculated from at least three independent experiments. Each experiment contained four parallel samples that were averaged. The standard deviations were calculated based on the average values for the independent experiments.

#### 3.2.8. Determination of Inhibitory Constants (K_i_) for Glycogen Phosphorylase

Enzyme activity in the direction of glycogen synthesis was assayed. Kinetic data were collected using the muscle phosphorylase b (dephosphorylated, GPb) isoform. Kinetic data for the inhibition of phosphorylase by glucose analogues were obtained in the presence of 10 μg/mL enzyme, varying concentrations of α-D-glucose-1-phosphate (4–40 mM), constant concentration (1%) of glycogen, and AMP (1 mM). Enzymatic activities were presented in the form of a double-reciprocal plot (Lineweaver-Burk). The plots were analysed by a non-linear data analysis program. The inhibitor constants (K_i_) were determined by secondary plots, replotting the slopes from the Lineweaver-Burk plot against the inhibitor concentrations. The means of standard errors for all calculated kinetic parameters averaged to less than 10% [78,79].

#### 3.2.9. Statistical Analyses

Protein expression measurements, cytotoxicity assays, and glucose uptake were repeated in at least 3 independent experiments. The data for these measurements are presented as means ± SE. Protein expression levels, cytotoxicity assay, and glucose uptake levels were compared using ANOVA with Fisher’s posthoc analysis. Statistical comparisons were performed using Statistica software (v.13.6.0, TIBCO Software, Palo Alto, CA, USA).

All IC_50_ and K_i_ calculations were prepared using the GraphPad Prism software. The data were plotted on a logarithmic scale as a function of inhibitor concentration. The IC_50_ values were calculated by non-linear regression analysis from sigmoidal dose-response curves. The results of kinetic experiments were calculated from at least three independent experiments. Each experiment contained four parallel samples that were averaged. The standard deviations were calculated based on the average values for the independent experiments.

## 4. Conclusions

New series of (*C*-β-D-glucopyranosylhetaryl)methyl benzene derivatives with 3-glucosyl-1,2,4- and 2-glucosyl-1,3,4-oxadiazole, 2-glucosylpyrimidine, and 2-glucosylimidazole skeletons were prepared. The deprotected compounds, together with some previously obtained 1-glucosyl-1,2,3-triazole and 3-glucosyl-1,2,4-triazole derivatives and known β-D-glucopyranosyl heterocyclic glycogen phosphorylase inhibitors, were assayed as SGLT1 and 2 inhibitors in a transfected TSA201 cell system. The (*C*-β-D-glucopyranosylhetaryl)methyl benzene type compounds showed low micromolar inhibition of SGLT2 in some cases, demonstrating that the structure of the proximal heterocyclic ring has a strong bearing on the inhibitory effect. On the other hand, none of these derivatives inhibited GP. Among the GP inhibitors, some low micromolar inhibitors of SGLT2 were found. The most efficient compound, 2-(β-D-glucopyranosyl)-4(5)-(2-naphthyl)-imidazole, is also the best glucose analogue inhibitor of GP known to date. This finding expands and proves the dual-target concept to the SGLT2-GP proteins as a potential antidiabetic strategy for the first time. Current computational and synthetic efforts are directed at fine-tuning the glucosyl-heterocyclic skeleton and its substitution pattern to find more efficient dual-target compounds.

## Figures and Tables

**Table 1:** Inhibitory effects (IC_50_ [nM]) of phlorizin and selected gliflozins against sodium dependent glucose cotransporter (SGLT2) (selectivity SGLT2/SGLT1).

Compound	Inh.	Ref.	Compound	Inh.	Ref.
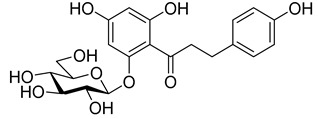 **1** Phlorizin	33 (7)	[19]	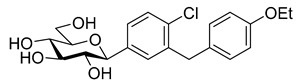 **2** Dapagliflozin (approved 2013: Forxiga^®^)	1.1 (1200)	[35]
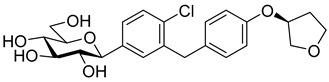 **3** Empagliflozin (approved 2014: Jardiance^®^)	3.1 (2677)	[36]	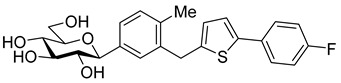 **4** Canagliflozin (approved 2013: Invokana^®^)	2.2 (414)	[37]
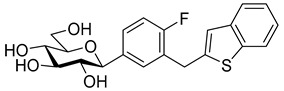 **5** Ipragliflozin (approved 2014: Suglat^®^)	7.4 (254)	[38]	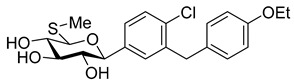 **6** Sotagliflozin (approved 2019: Zynquista^®^)	1.8 (20)	[34]

**Table 2:** Inhibitory potencies (IC_50_ [µM] *^a^*) of the new and some previously prepared compounds against SGLT1, SGLT2, and rabbit muscle glycogen phosphorylase b (rmGPb).

	Compound	SGLT2	SGLT1	rmGPb
**1**	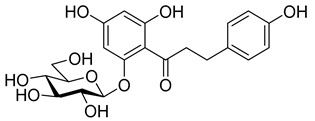	6.40 ± 1.12 *^b^*	131.17 ± 24.56 *^b^*	450 mM (IC_50_)
**2**	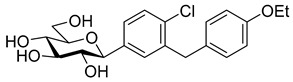	0.00748 ± 0.00182 *^b^*	0.83 ± 0.26 *^b^*	NI *^d^*
**10a**	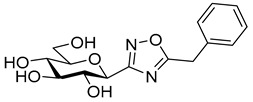	3.68 ± 0.59	20.72 ± 7.66	NI *^d^*
**10b**	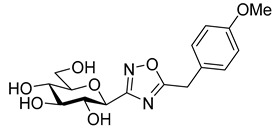	NI *^c^*	NI*^c^*	NI *^d^*
**10c**	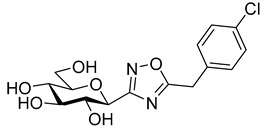	NI *^c^*	NI*^c^*	NI *^d^*
**13a**	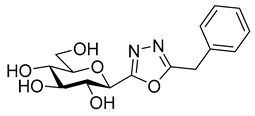	2.21 ± 0.58	23.72 ± 6.14	NI *^d^*
**13b**	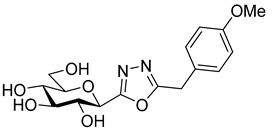	NI *^c^*	NI*^c^*	NI *^d^*
**13c**	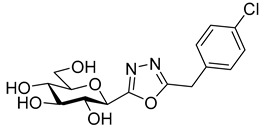	NI *^c^*	NI *^c^*	NI *^d^*
	Compound	SGLT2	SGLT1	rmGPb
**16a**	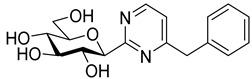	17.05 ± 3.16	17.98 ± 3.92	NI *^d^*
**16b**	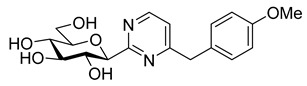	11.59 ± 3.19	NI*^c^*	NI *^d^*
**16c**	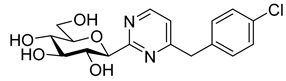	NI *^c^*	NI *^c^*	NI *^d^*
**16d**	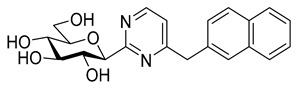	NI*^c^*	NI *^c^*	NI *^d^*
**18**	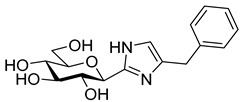	3.57 ± 0.42	11.7 ± 2.48	12% at 625 µM
**19a***^e^* [67]	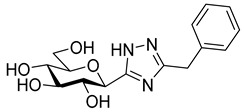	71.67 ± 10.64	19.59 ± 7.26	NI *^d^*
**19b***^e^* [67]	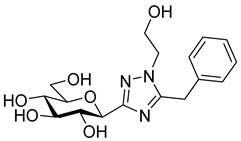	NI *^c^*	NI*^c^*	NI *^d^*
**19c***^e^* [67]	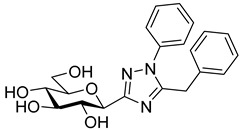	2.44 ± 0.85	35.50 ± 9.56	NI *^d^*
**20***^e^* [68]	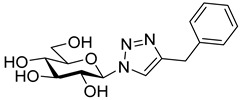	23.21 ± 1.86	101.45 ± 23.46	600 µM (IC_50_) [68]

*^a^* The inhibition of SGLT1 and SGLT2 was measured in TSA-201 cells. *^b^* The IC_50_ values of compounds **1-2** were used as controls for our in-house assay. *^c^* NI: no inhibition at 1000 µM. *^d^* NI: no inhibition at 625 µM. *^e^*The synthesis of the compound is described in the cited reference.

**Table 3:** Inhibitory potencies (IC_50_ [µM] *^a^*) of some aryl-substituted glucopyranosyl azole type glycogen phosphorylase inhibitors*^b^* against SGLT2 and SGLT1.

	Compound	SGLT2	SGLT1	rmGPb*^b^*(K_i_ [µM])
**21a**	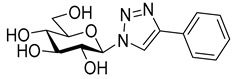	67.28 ± 10.90	167.07 ± 63.50	151 [69] 162 [68]
**21b**	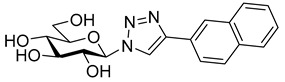	10.34 ± 2.18	126.90 ± 21.55	16 [69] 36 [68]
**22a**	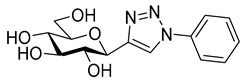	9.10 ± 2.94	242.13 ± 37.91	NI*^d^* [70]
**22b**	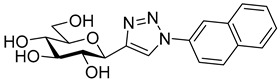	111.31 ± 27.56	23.77 ± 5.72	NI*^d^* [70]
**23a**	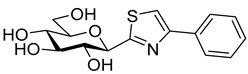	NI*^c^*	NI*^c^*	310 [71]
**23b**	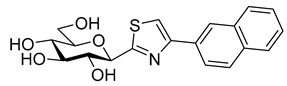	NI*^c^*	NI*^c^*	158 [71]
**24a**	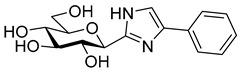	91.04 ± 0.56	244.60 ± 42.02	0.28 [71]
**24b**	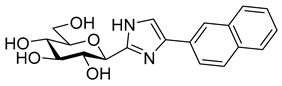	3.50 ± 0.88	11.89 ± 2.61	0.031 [71]
**25a**	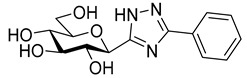	15.01 ± 2.74	124.56 ± 46.51	7 [72,73]
**25b**	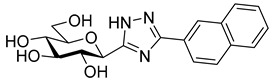	17.72 ± 2.32	12.98 ± 2.88	0.41 [72,73]

*^a^* The inhibition of SGLT2 and SGLT1 was measured in TSA-201 cells. *^b^* rmGPb: rabbit muscle glycogen phosphorylase b. The synthesis of the compounds is also described in the cited references. *^c^* NI: no inhibition at 1000 µM. *^d^* NI: no inhibition at 625 µM.

## Data Availability

Not applicable.

## Supplementary Materials

The following are available online at https://www.mdpi.com/article/10.3390/ph14040364/s1, Figure S1: Western blot analysis of SGLT1 and GAPDH protein in TSA, transfected TSA201 cells, and mouse kidney, Figure S2: Western blot analysis of SGLT2 and GAPDH protein in TSA, transfected TSA201 cells, and mouse kidney, Figure S3: Representative dose-response curves for calculating IC_50_ values.
