# Supplementary material for: Dual-Target Compounds against Type 2 Diabetes Mellitus: Proof of Concept for Sodium Dependent Glucose Transporter (SGLT) and Glycogen Phosphorylase (GP) Inhibitors
Source: Pharmaceuticals (Basel). 2021 Apr 15;14(4):364. doi: 10.3390/ph14040364 (PMC8071193; doi:10.3390/ph14040364)
Supplement: Supplementary file 1 [file pharmaceuticals-14-00364-s001.zip › pharmaceuticals-1142811-supplementary.pdf]

Supplementary Information

Dual-Target Compounds against Type 2 *Diabetes Mellitus*: Proof of Concept for Sodium Dependent Glucose Transporter (SGLT) and Glycogen Phosphorylase (GP) Inhibitors

Ádám Sipos [1,2], Eszter Szennyes [3], Nikolett Éva Hajnal [3], Sándor Kun [3], Katalin E. Szabó [3], Karen Uray [1], László Somsák [3,*], Tibor Docsa [1,*] and Éva Bokor [3,*]

1 Department of Medical Chemistry, Faculty of Medicine, University of Debrecen, Egyetem tér 1, H-4032 Debrecen, Hungary; sipos.adam@med.unideb.hu (Á.S.); karen.uray@med.unideb.hu (K.U)
2 Doctoral School of Molecular Medicine, University of Debrecen, Egyetem tér 1, H-4032 Debrecen, Hungary
3 Department of Organic Chemistry, University of Debrecen, H-4002 Debrecen, POB 400, Hungary; szeszterke11@gmail.com (E.Sz.); hajnalnikoletteva@gmail.com (N.É.H.); kun.sandor@science.unideb.hu (S.K.); szabo.erzsebet.katalin@science.unideb.hu (K.E.Sz.)
* Correspondence: somsak.laszlo@science.unideb.hu; Tel.: + 36-525-129-00 ext 22348 (L.S.); tdocsa@med.unideb.hu; Tel.: + 36-525-186-00 ext 61192 (T.D.); bokor.eva@science.unideb.hu; Tel.: + 36-525-129-00 ext 22474 (É.B.)

Full Western blot pictures

Representative dose-response curves for calculating IC_{50} values.

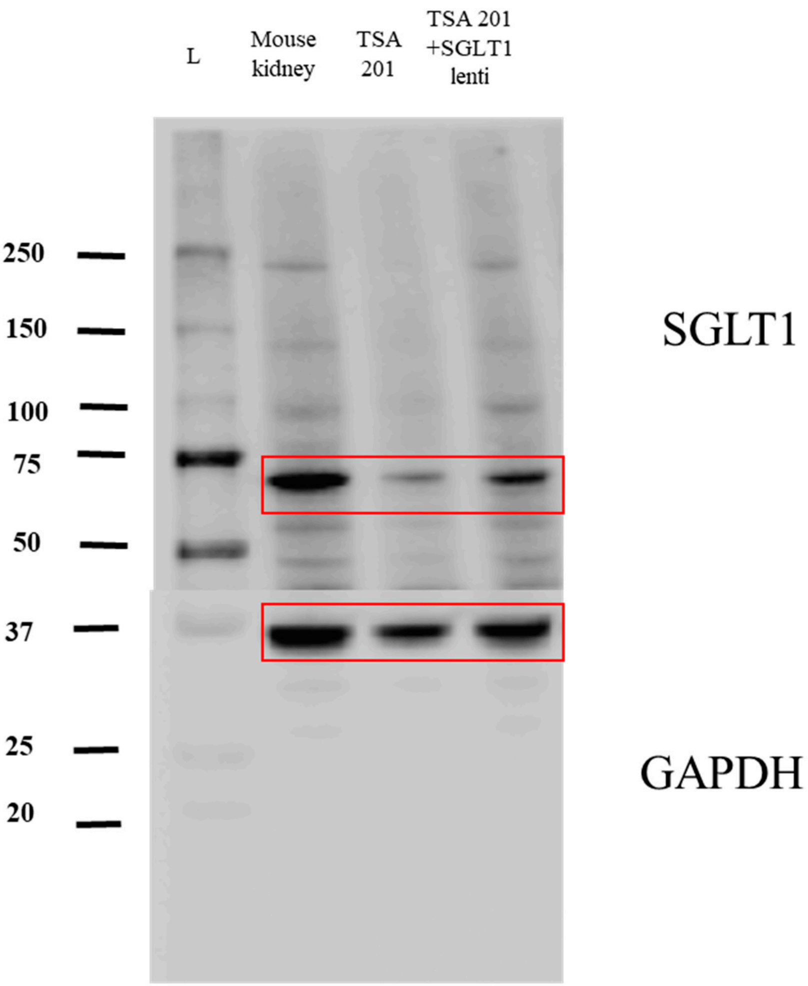

Figure S1. Western blot analysis of SGLT1 and GAPDH protein in TSA, transfected TSA201 cells, and mouse kidney. The membrane was incubated with anti-SGLT1 (Origene anti-SLC5A1 TA324226) or anti-GAPDH (EMD Millipore, ABS16) antibodies in 1% BSA solution. Super Signal West Femto and Pico Maximum Sensitivity Substrates (Sigma-Aldrich) were used to detect antibody binding. These are full blot images. GAPDH was used to normalize for protein loading. The results were quantified by BIORAD Image Lab 5.2.1 software. Protein load: 20 mg/lane. L, Molecular Weight Protein Ladder.

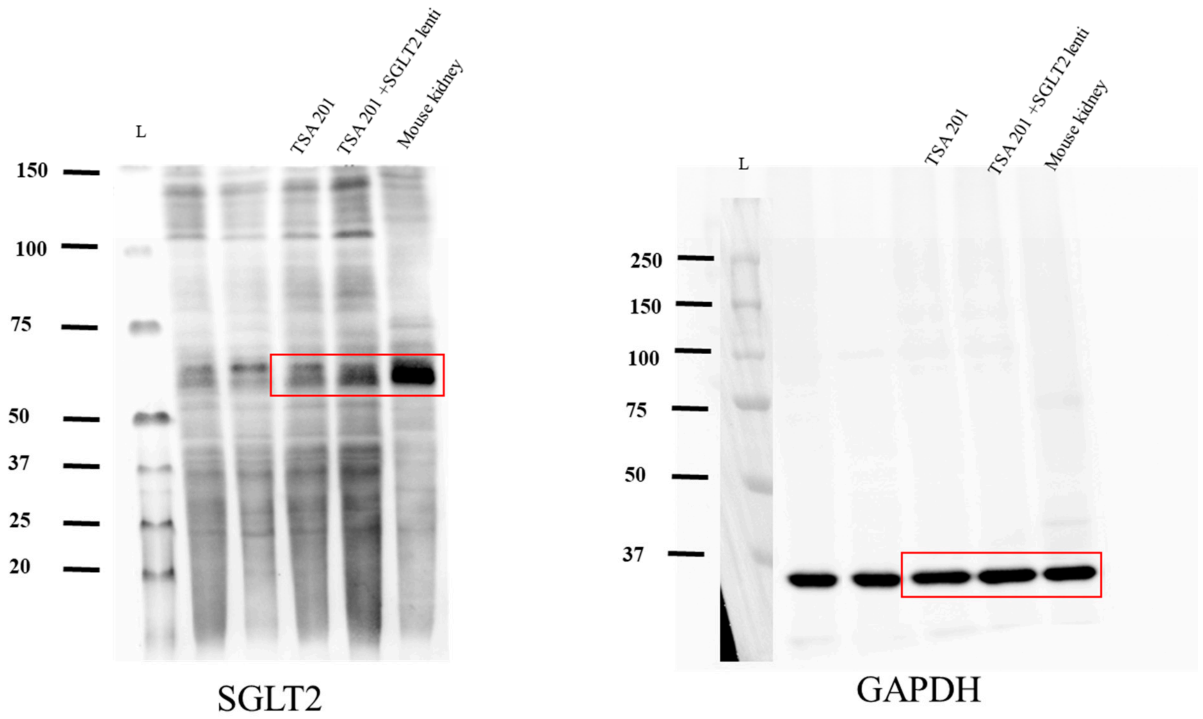

Figure S2. Western blot analysis of SGLT2 and GAPDH protein in TSA, transfected TSA201 cells, and mouse kidney. The membranes were incubated with anti-SGLT2 (Abcam, ab137207) or anti-GAPDH (EMD Millipore, ABS16) antibodies in 1% BSA solution. Super Signal West Femto and Pico Maximum Sensitivity Substrates (Sigma-Aldrich) were used to detect antibody binding. These are full blot images. GAPDH was used to normalize for protein loading. The results were quantified by BIORAD Image Lab 5.2.1 software. Protein load: 20 mg/lane. L, Molecular Weight Protein Ladder.

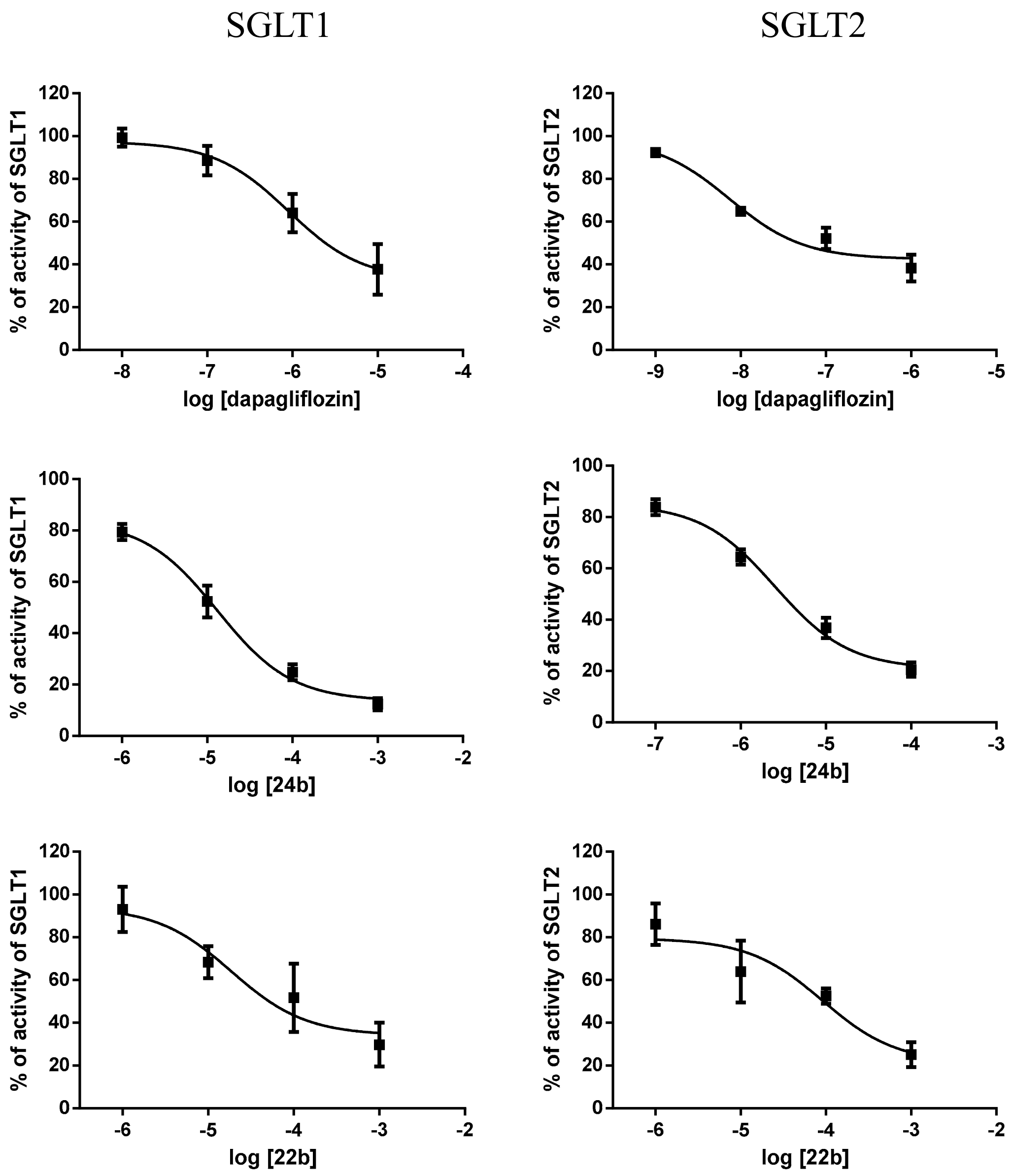

Figure S3. Representative dose-response curves for calculating IC_{50} values of dapagliflozin, compound **24b**, and compound **22b** for the inhibition of SGLT1 and 2. All calculations were prepared using the GraphPad Prism software. The data were plotted on a logarithmic scale as a function of inhibitor concentration. The IC_{50} values were calculated using non-linear regression analysis from the sigmoidal dose-response curves. Each experiment was repeated a minimum of three times.
